# Development and Assessment of a Model for Predicting Individualized Outcomes in Patients With Oropharyngeal Cancer

**DOI:** 10.1001/jamanetworkopen.2021.20055

**Published:** 2021-08-09

**Authors:** Lauren J. Beesley, Andrew G. Shuman, Michelle L. Mierzwa, Emily L. Bellile, Benjamin S. Rosen, Keith A. Casper, Mohannad Ibrahim, Sarah M. Dermody, Gregory T. Wolf, Steven B. Chinn, Matthew E. Spector, Robert J. Baatenburg de Jong, Emilie A. C. Dronkers, Jeremy M. G. Taylor

**Affiliations:** 1 Department of Biostatistics, University of Michigan, Ann Arbor; 2 Department of Otolaryngology–Head and Neck Surgery, University of Michigan, Ann Arbor; 3 Department of Radiation Oncology, University of Michigan, Ann Arbor; 4 Department of Radiology, University of Michigan, Ann Arbor; 5 Department of Otorhinolaryngology–Head and Neck Surgery, Erasmus Cancer Institute, Erasmus University Medical Center, Rotterdam, the Netherlands

## Abstract

**Importance:**

Recent insights into the biologic characteristics and treatment of oropharyngeal cancer may help inform improvements in prognostic modeling. A bayesian multistate model incorporates sophisticated statistical techniques to provide individualized predictions of survival and recurrence outcomes for patients with newly diagnosed oropharyngeal cancer.

**Objective:**

To develop a model for individualized survival, locoregional recurrence, and distant metastasis prognostication for patients with newly diagnosed oropharyngeal cancer, incorporating clinical, oncologic, and imaging data.

**Design, Setting, and Participants:**

In this prognostic study, a data set was used comprising 840 patients with newly diagnosed oropharyngeal cancer treated at a National Cancer Institute–designated center between January 2003 and August 2016; analysis was performed between January 2019 and June 2020. Using these data, a bayesian multistate model was developed that can be used to obtain individualized predictions. The prognostic performance of the model was validated using data from 447 patients treated for oropharyngeal cancer at Erasmus Medical Center in the Netherlands.

**Exposures:**

Clinical/oncologic factors and imaging biomarkers collected at or before initiation of first-line therapy.

**Main Outcomes and Measures:**

Overall survival, locoregional recurrence, and distant metastasis after first-line cancer treatment.

**Results:**

Of the 840 patients included in the National Cancer Institute–designated center, 715 (85.1%) were men and 268 (31.9%) were current smokers. The Erasmus Medical Center cohort comprised 300 (67.1%) men, with 350 (78.3%) current smokers. Model predictions for 5-year overall survival demonstrated good discrimination, with area under the curve values of 0.81 for the model with and 0.78 for the model without imaging variables. Application of the model without imaging data in the independent Dutch validation cohort resulted in an area under the curve of 0.75. This model possesses good calibration and stratifies patients well in terms of likely outcomes among many competing events.

**Conclusions and Relevance:**

In this prognostic study, a multistate model of oropharyngeal cancer incorporating imaging biomarkers appeared to estimate and discriminate locoregional recurrence from distant metastases. Providing personalized predictions of multiple outcomes increases the information available for patients and clinicians. The web-based application designed in this study may serve as a useful tool for generating predictions and visualizing likely outcomes for a specific patient.

## Key Points

**Question:**

Can a model be developed for individualized survival, locoregional recurrence, and distant metastasis prognostication for patients newly diagnosed with oropharyngeal cancer, incorporating clinical, oncologic, and imaging data?

**Findings:**

In this prognostic study, model predictions for 5-year overall survival demonstrated excellent discrimination in cohort study training data for models with and without imaging variables. This model appeared to possess good calibration and well stratified patients in terms of likely outcomes among many competing events.

**Meaning:**

This prognostic model and web-based application may serve as a useful tool to provide clinicians, researchers, and patients with oropharyngeal cancer robust, individualized prognostic estimations.

## Teaser

This prognostic study reports on the development of a model including factors used in establishing an individualized prognosis for locoregional recurrence, distant metastasis, and survival in patients with oropharyngeal cancer.

## Introduction

With the increasing prevalence of human papillomavirus (HPV)–associated disease, our understanding of the biologic characteristics and treatment of oropharyngeal squamous cell carcinoma (OPSCC) has evolved. Patients with HPV-associated cancers tend to be younger and healthier than patients with HPV-negative cancers.^1,2^ The American Joint Committee on Cancer (AJCC) 8th edition staging system directly incorporates HPV status (using p16 expression as a surrogate) as the first biologic marker to be included in clinical head and neck cancer staging.^3^ Multiple national studies are under way to evaluate treatment de-escalation in selected patients with HPV-positive OPSCC.^1,2,4,5,6,7,8^ Data suggest that patterns of recurrence may differ by HPV status, with higher rates of local recurrence and worse disease-specific and overall survival among patients with HPV-negative tumors.^9,10^

Individualized prognostic calculators, such as nomograms, are important tools for evaluating risk. Unlike traditional staging systems that place patients in broad risk categories, prognostic calculators can provide the probability of a future event (eg, cancer recurrence or death) given patients’ individual characteristics. In previous work,^11^ 4 different prognostic calculators for OPSCC overall survival were evaluated.^9,12,13,14^ Other earlier work^15,16^ identified substantial differences in predicted overall survival for the same patient between calculators. This discrepancy is partially explained by training data sets of limited size and models with fewer risk factors. Thus, there is a need for improved models that include multiple competing risk factors and are based on high-quality, large databases.

Many existing prognostic calculators for oropharyngeal cancer estimate overall or recurrence-free survival probabilities.^9,17^ However, clinicians and patients may be interested in a more-detailed characterization of patients’ risk for treatment decision making. For example, there is interest in the probability of death due to cancer vs noncancer causes, as well as separating recurrence by type (eg, locoregional vs distant). Prognostic calculators that can generate predictions for multiple competing outcomes provide a clearer picture of patients’ disease landscape and potential trajectory.

In addition to standard factors, such as T and N classification, literature suggests that imaging biomarkers may be prognostically useful, particularly for distinguishing between the likelihood of locoregional and distant recurrence.^18,19,20,21,22,23^ Imaging biomarkers could inform treatment decisions and trial candidacy.

**Figure 1.**
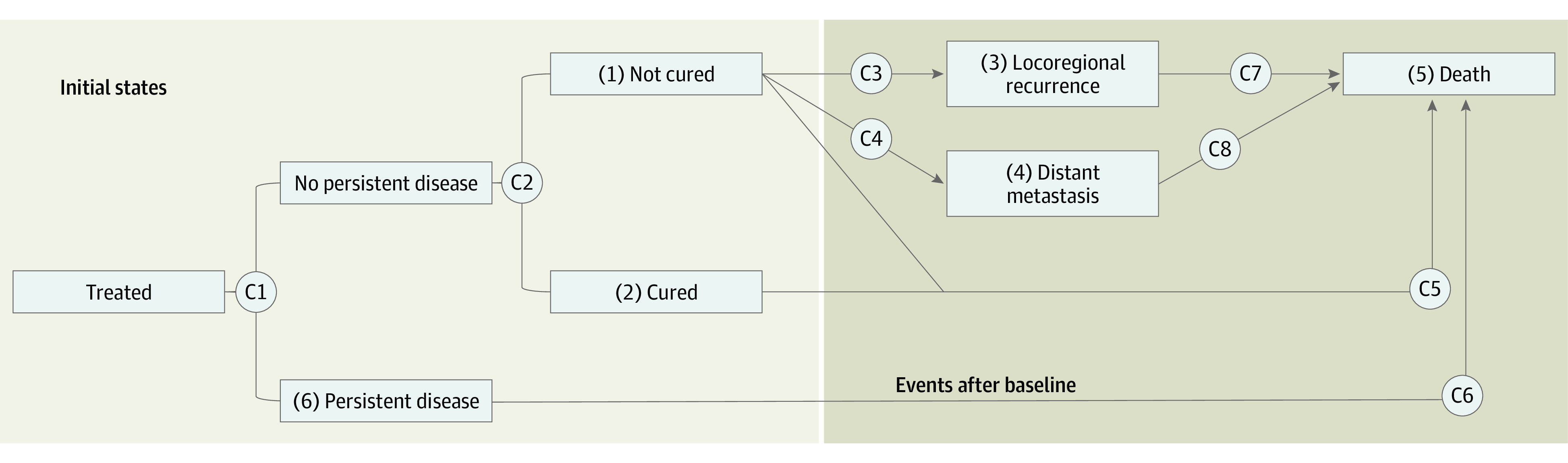
Multistate Model of Recurrence and Death in Patients With Oropharyngeal Cancer The multistate model is composed of 8 component models (C1-C8). Boxes correspond to 3 initial states (states 1, 2, and 6) and 3 outcome event states (states 3, 4, and 5). Brackets correspond to 2 logistic regression models related to initial state (C1 and C2). Arrows correspond to proportional hazards regression models for each of the following events: locoregional recurrence for patients who are not cured (C3: states 1 to >3), distant metastasis for patients who are not cured (C4: states 1 to >4), death without previous recurrence for patients without persistent disease (C5: states 1 or 2 to state 5), death among patients with persistent disease (C6: state 6 to state 5), death after locoregional recurrence (C7: state 3 to state 5), and death after distant metastasis (C8: state 4 to state 5).

To address this need, we developed a prognostic calculator for OPSCC that can provide detailed predictions of recurrence and survival outcomes based on each patient’s individual characteristics, augmented with imaging biomarkers. Predictions are based on a bayesian multistate model (Figure 1) that structurally incorporates the possible patterns of progression after treatment, including the possibilities of being cured or having persistent disease after initial therapy.^24,25^ We hypothesized that this multistate model-based prognostic calculator combining clinical, oncologic, and imaging data can provide clinicians and patients with robust, individualized predictions that may facilitate cancer treatment decision making and counseling.

## Methods

We considered data from 840 patients diagnosed with OPSCC at the University of Michigan (UM) between January 2003 and August 2016; analysis was performed between January 2019 and June 2020. Extensive patient information was collected at diagnosis, and patients were followed up prospectively. Patient data collection, extraction, and analysis were approved by a UM institutional review board. All patients provide written informed consent; there was no financial compensation. The Table provides summary information for this cohort. Additional information has been published.^11,26,27,28^ This study followed the Transparent Reporting of a Multivariable Prediction Model for Individual Prognosis or Diagnosis (TRIPOD) reporting guideline for prognostic studies.

**Table.** Characteristics of Patients With Oropharyngeal Cancer Treated at the University of Michigan and Treated at Erasmus Medical Center

Characteristic	No. (%)	
	University of Michigan (n = 840)	Erasmus University Medical Cancer (n = 447)
Age at diagnosis, median (IQR), y	58.0 (52-64.4)	59.9 (53.7-67.8)
Sex		
Male	715 (85.1)	300 (67.1)
Female	125 (14.9)	147 (32.9)
Anemia^a^		
No	597 (71.1)	214 (47.9)
Yes	125 (14.9)	107 (23.9)
Unknown	118 (14.0)	126 (28.2)
ACE27 comorbidity		
None	205 (24.4)	171 (38.3)
Mild	257 (30.6)	137 (30.6)
Moderate	111 (13.2)	106 (23.7)
Severe	44 (5.2)	31 (6.9)
Unknown	223 (26.5)	2 (0.5)
Smoking status		
Never	277 (33.0)	35 (7.8)
Former	290 (34.5)	54 (12.1)
Current	268 (31.9)	350 (78.3)
Unknown	5 (0.6)	8 (1.8)
T classification (AJCC 8th edition)		
1	189 (22.5)	47 (10.5)
2	279 (33.2)	139 (31.1)
3	136 (16.2)	156 (34.9)
4	233 (27.7)	104 (23.3)
Unknown	3 (0.4)	1 (0.2)
N classification (AJCC 8th edition)		
0	100 (11.9)	145 (32.4)
1	380 (45.2)	107 (23.9)
2	176 (21.0)	162 (36.2)
3	77 (9.2)	30 (6.7)
Unknown	107 (12.7)	3 (0.7)
p16		
Negative	88 (10.5)	363 (81.2)
Positive	425 (50.6)	84 (18.8)
Unknown	327 (38.9)	0
Metabolic tumor volume, median (IQR), mL	13.6 (8.4-20.7)	NA
Unknown	564 (67.1)	NA
Radiologic extracapsular extension		
No	222 (26.4)	NA
Yes	54 (6.5)	NA
Unknown	564 (67.1)	NA
Treatment modality		
Chemoradiotherapy	648 (77.1)	122 (27.3)
Chemotherapy alone	14 (1.7)	0
Radiotherapy alone	40 (4.8)	122 (27.3)
Surgery (with or without adjuvant therapy)	83 (9.9)	165 (36.9)
Unknown	55 (6.5)	38 (8.5)

Abbreviations: ACE27, Adult Comorbidity Evaluation 27; AJCC, American Joint Committee on Cancer; IQR, interquartile range; NA, not applicable.

^a^ Defined as hemoglobin less than 12 g/dL for women and less than 13 g/dL for men (to convert to grams per liter, multiply by 10).

### Outcomes and Cohorts

The primary outcomes were locoregional recurrence, distant metastases, and death. Death and recurrence information was collected prospectively and supplemented by medical record abstraction and official death records. Locoregional recurrence was defined as biopsy-proven or clinically overt imaging identification of cancer recurrence at the primary tumor site or cervical lymph nodes, and distant metastasis was defined as the identification of metastasis outside the head and neck by biopsy or clinically overt imaging. Suspected second primary tumors were excluded. The survival outcome was defined as the minimum time from diagnosis to the date of death, loss to follow-up, or March 18, 2019. Patients with observable cancer posttreatment (including newly detected metastasis) at or before routine 12-week posttreatment scans were defined as having persistent disease. Among patients with less than 3 months’ follow-up for recurrence, those who died within 6 months of diagnosis were also listed as having persistent disease.

Baseline covariates in the UM cohort included age at diagnosis, sex, Adult Comorbidity Evaluation 27 score (none, mild, moderate, and severe), smoking habits (never, former, and current in last 12 months), anemia (yes or no), p16 status (positive or negative), clinical T classification (AJCC 8th edition: T1, T2, T3, and T4), and clinical N classification (AJCC 8th edition: N0, N1, N2abc, and N3). We defined anemia as hemoglobin level less than 12 g/dL for women and 13 g/dL for men (to convert to grams per liter, multiply by 10). For 276 patients, pretreatment fluorodeoxyglucose positron emission tomography and diagnostic computed tomography scans were available. We defined metabolic tumor volume (MTV) as the volume of total tumor burden with standardized uptake value greater than 50% of the maximum value analyzed as a continuous variable. We defined radiologic extracapsular extension (rECE) as a binary indicator of overt haziness of the lymph node capsule and/or lack of discernible fat plane between a lymph node and the sternocleidomastoid muscle, and an equivocal rECE finding was considered negative.^20,21^

Data from 447 patients treated for OPSCC between January 2000 and December 2006 at Erasmus Medical Center (MC) in Rotterdam, the Netherlands, were used for external validation. Patients were identified through the Dutch Cancer Registries, and patient information was obtained retrospectively from patient medical records. Pretreatment radiographic metrics were unavailable. A cohort description is given by Rietbergen et al^29,30^ and in the Table.

### Statistical Analysis

Cancer recurrence and survival were described by a multistate model composed of 8 component models (Figure 1), each of which may depend on baseline covariates. The multistate model structure was determined based on clinical outcome patterns for patients with OPSCC.^24,25^ Patients were assumed to be in 1 of 3 initial states, defined shortly after treatment. Some patients treated for their primary cancer can be cured and will never experience a primary cancer recurrence (state 2),^31,32,33^ some patients will have persistent disease (state 6), and other patients will have a complete response to initial therapy but cancer will recur (state 1). These 3 groups of patients were expected to have different prognoses and were modeled separately. Initial state was assumed to take a fixed, possibly unknown, value for each patient, and 2 logistic regression models (1 for the probability of being persistent [component model 1] and 1 for the probability of being noncured if nonpersistent [component 2], each depending on baseline covariates) were used to model these 3 states.

After treatment, we considered 3 outcome events: locoregional recurrence (state 3, with or without subsequent metastasis), distant metastasis (state 4, with or without subsequent locoregional recurrence), and death (state 5). Each arrow in Figure 1 corresponds to a possible event transition. The hazard rate for each transition was assumed to follow a proportional hazards form depending on baseline covariates, with time measured in months (components 3-8).

A subset of baseline covariates was incorporated in each component model, where the subset was chosen based on prior beliefs and exploratory analyses. We did not allow for covariate effects in the model for death among patients with persistent disease (component 6), because these events occurred quickly. Weibull baseline hazards were assumed for all transitions except for transitions to locoregional recurrence or distant metastasis (components 3 and 4), for which we assumed piecewise Weibull hazards with change points at 6 months. For modeling death after recurrence (components 7 and 8), we used a clock-reset approach in which the transition time to death was defined in months since entering the recurrence state.^34^ eAppendix 1, eAppendix 2, and eTable 1 in the Supplement provide the exact model structure. We considered 2 model formulations, with the second incorporating the imaging variables MTV and rECE. We extended a multistate model in Beesley et al^35^ and Conlon et al^36^ to incorporate persistence and recurrence subtypes.

Bayesian estimation was performed using a custom Markov Chain Monte Carlo algorithm in R (R Foundation) run for 25 000 iterations with a burn-in of 10 000 iterations. We incorporated prior ordering information for some associations (eg, increasing risk of metastasis for higher N classification). Missing values were imputed during estimation using a chained equations procedure.^37,38,39^ Additional details are given in eAppendix 3 and eTable 6 in the Supplement. Prior distributions are provided in eTable 3 and eTable 4 in the Supplement.

We used the model structure and posterior means of model parameters to predict the likelihood of different outcomes for individual patients. For example, we could predict the proportion of patients with certain baseline characteristics who will be event-free, alive with recurrent cancer, or dead at 5 years, called *state occupancy probabilities* (formulas in eAppendix 4 in the Supplement). These probabilities correspond to each possible sequence of events patients may experience after treatment up to a given time.

Validation involved comparing observed and predicted outcomes in UM and Erasmus MC data. We evaluated predicted state occupancy probabilities using the following metrics: (1) discrimination using area under the receiver operating curves (AUC) for 5-year overall and event-free survival, (2) goodness-of-fit using Cox-Snell diagnostics, and (3) calibration comparing Kaplan-Meier estimates of observed event rates with predicted probabilities.^11,40^ We also compared predictions over time (averaged across patients) with estimated Kaplan-Meier and cumulative incidence curves. For patients with missing data, we obtained each patient’s predictions as their average prediction across 10 imputed data sets. Predicted outcomes are independent of initial treatment modality.

## Results

Of the 840 UM patients diagnosed with oropharyngeal cancer in this study, 47 (5.6%) patients were observed to have persistent disease after initial treatment, 185 (22.0%) had an observed recurrence, 186 (22.1%) had at least 72 months of follow-up without recurrence (defined as being cured of their primary disease), and 272 (32.4%) died. A total of 715 (85.1%) patients were male and 268 (31.9%) were current smokers.

Of the 447 patients in the Erasmus MC validation cohort, 38 (8.5%) patients were observed to have persistent disease after initial treatment, 114 (25.5%) had observed recurrence, 96 (21.5%) had at least 72 months of follow-up without recurrence, and 310 (69.4%) died. A total of 300 (67.1%) patients were male and 350 (78.3%) were current smokers. The Table and eTable 1 and eTable 2 in the Supplement describe the UM and Erasmus MC patients.

**Figure 2.**
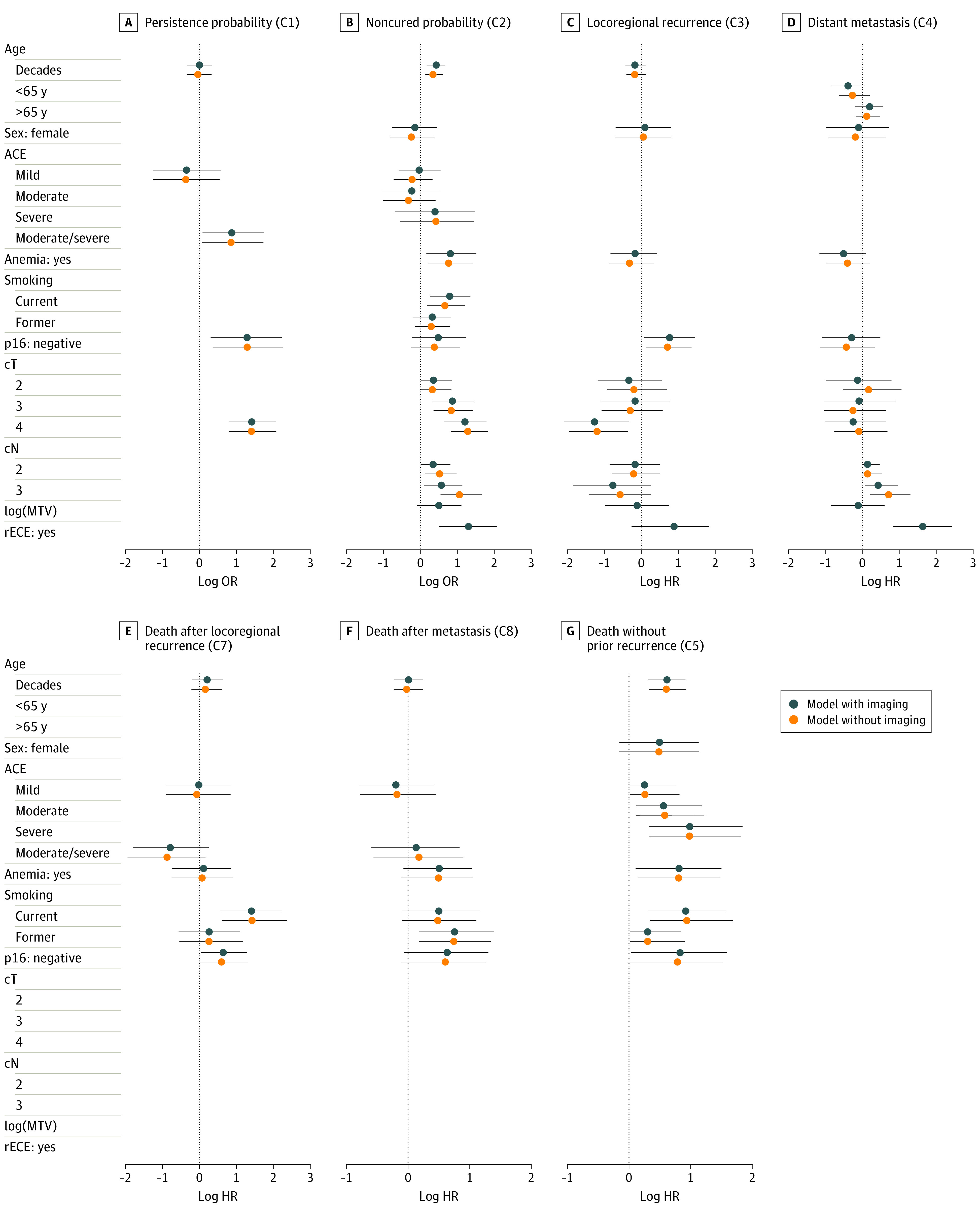
Estimated Covariate Associations and 95% Credible Intervals for Bayesian Multistate Model Fits Results for a model with and without metabolic tumor volume (MTV) and radiologic extracapsular extension (rECE) are shown. Panels A to G correspond to a component (C) model of the larger multistate model. Panels C to G correspond to log hazard ratios (HRs) for event transition models (components 3-8), and panels A and B correspond to log odds ratios (ORs) from the logistic regression models for the initial state (components 1 and 2). Reference categories include male sex, no Adult Comorbidity Evaluation (ACE) 27 comorbidities, no anemia, never smoker, p16-positive, cT1 classification, cN0 or cN1 classification, and no rECE. Dashed lines distinguish parameters representing different covariates. Error bars indicate 95% credible intervals for bayesian multistate model fits.

Figure 2 shows the posterior means and 95% credible intervals (CrIs) for covariate associations in each component of the multistate model. eTable 5 in the Supplement provides estimates for baseline hazard and intercept parameters. Results for the model with and without MTV and rECE are shown, which tend to be similar. logMTV and rECE were both associated with higher probabilities of being noncured among patients with nonpersistent disease (logMTV: odds ratio, 1.65; 95% CrI, 0.91-3.07; rECE: odds ratio, 3.67; 95% CrI, 1.677.88). rECE was also associated with the rates of locoregional recurrence and metastasis among patients who were not cured (locoregional recurrence: hazard ratio, 2.42; 95% CrI, 0.77-6.26; metastasis: hazard ratio, 5.11; 95% CrI, 2.32-11.24). Smoking status, cT and cN category, age, and anemia were all associated with higher probabilities of noncure among patients with initial complete responses to therapy. Furthermore, higher cT and cN categories in these patients were associated with lower rates of locoregional recurrence. Worse Adult Comorbidity Evaluation 27 comorbidities, cT4 category, and p16-negative tumors were all associated with higher probabilities of persistent disease. In addition, p16-negative cancers were associated with a higher rate of locoregional recurrence and death with or without earlier recurrence. eFigure 5 in the Supplement provides estimates for other model parameters.

### Evaluation of Individualized Predictions

We applied the formulas given in eAppendix 4 in the Supplement to calculate predicted event probabilities over time for each patient in the UM cohort. We then compared these predictions with observed outcomes in eAppendix 5 in the Supplement (eFigures 1-5 in the Supplement). Predictions for 5-year overall survival demonstrated good discrimination in the UM data, with AUC values of 0.81 for the model with and 0.78 for the model without imaging variables. For comparison, the AJCC 8th edition overall cancer stage had an AUC of 0.71 in our data, and the Larsen et al^12^ model produced an AUC of 0.78 (eTable 7 in the Supplement).

**Figure 3.**
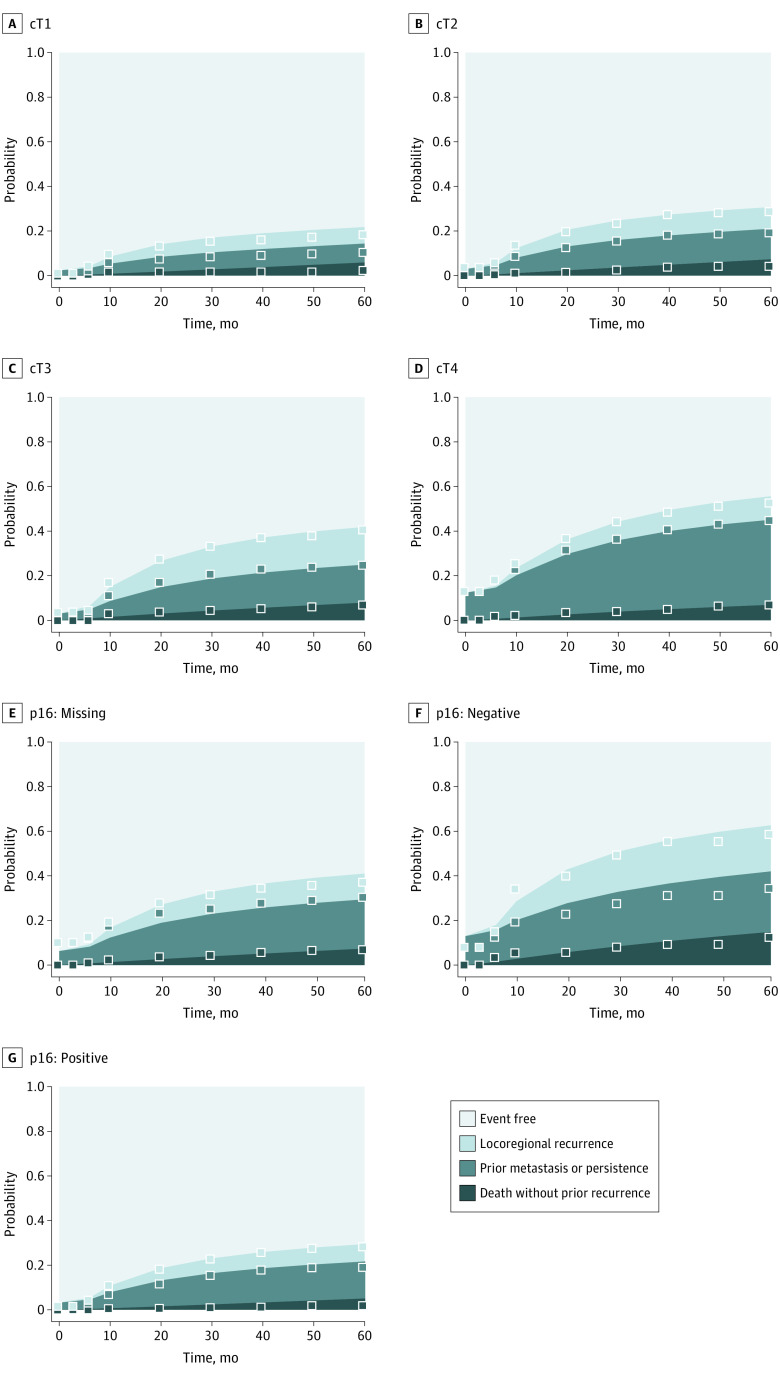
Calibration of Predictions With Observed Outcomes Over Time Predictions shown for cT classification (A-D) and p16 status (E-G) indicated for the average model-based prediction (model without imaging variables). These predictions were broken into 4 possible outcomes: (1) no event, (2) alive or died with previous metastasis as the first event, (3) alive or died with previous locoregional recurrence as the first event or with persistent disease, and (4) death without previous recurrence or persistent disease. By definition, these predicted probabilities sum to 1 at each time horizon. Plotted points indicate the corresponding cumulative incidence estimate for outcomes 1, 1 or 2, and 1 or 2 or 3, based on observed outcome data for people with the corresponding cT or p16 status.

In Figure 3, we compare model predictions (averaged across patients) to estimated cumulative incidence curves with strata defined by cT classification and p16 status. We observed similarity between observed and predicted event rates, indicating good calibration and an ability to stratify patients in terms of likely outcomes among many competing events.

In the Erasmus MC validation cohort, the AUC estimates for the model without imaging variables were 0.75 for 5-year overall survival and 0.72 for 5-year event-free survival (eAppendix 6 in the Supplement). C-indices were 0.70 and 0.69. eFigure 6 in the Supplement shows calibration of predicted 5-year overall and event-free survival probabilities to Kaplan-Meier estimates, indicating good calibration at 5 years for both outcomes. Calibration of state occupancy predictions over time is shown in eFigure 7 in the Supplement, and eFigure 8 in the Supplement provides Cox-Snell diagnostics. Results indicated substantial underestimation of mortality without earlier recurrence relative to observed outcomes before 5 years.

**Figure 4.**
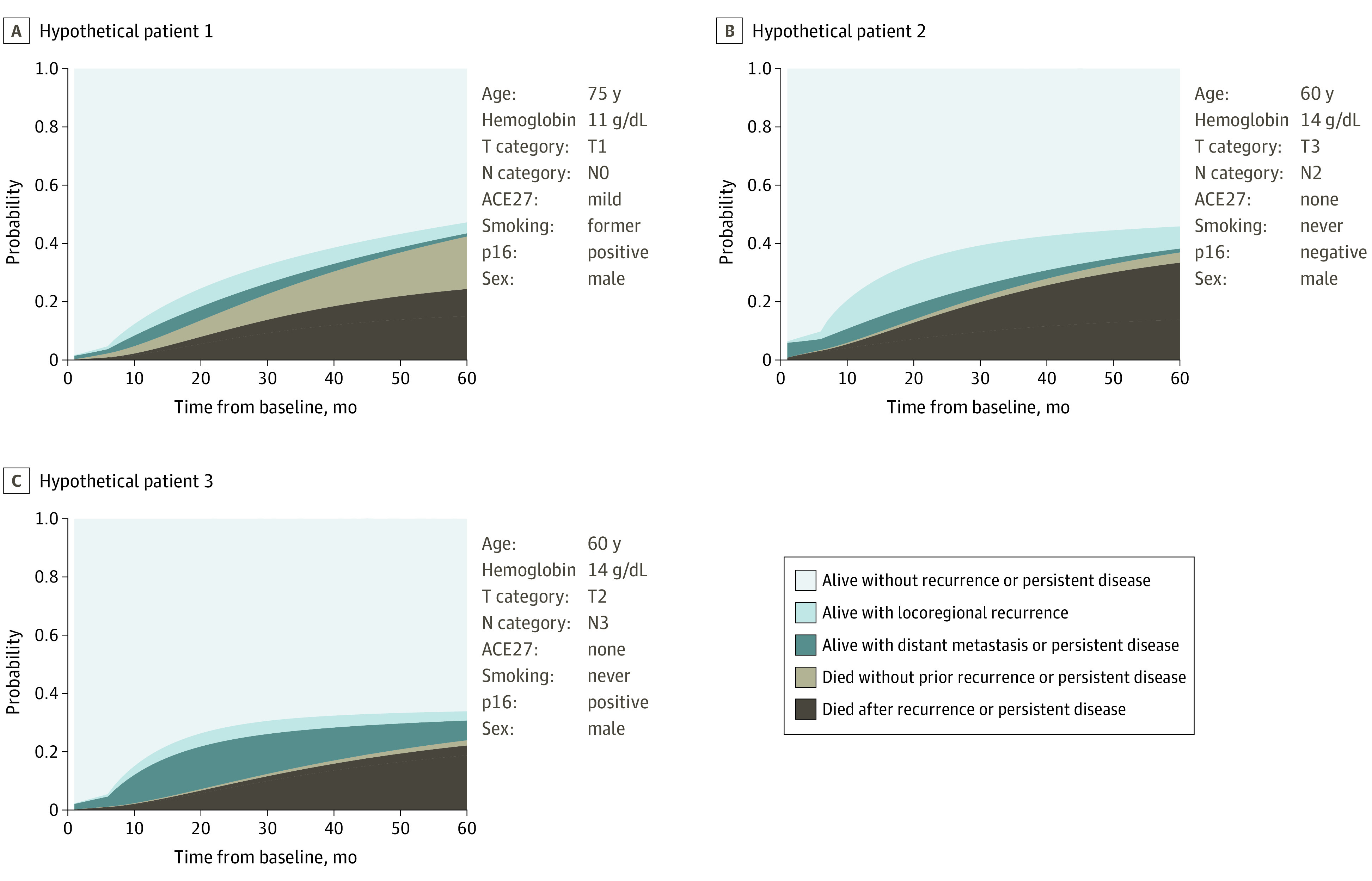
Individual Predictions for 3 Hypothetical Patients Using Model Without Imaging Biomarkers To convert hemoglobin to grams per liter, multiply by 10. ACE27 indicates Adult Comorbidity Evaluation 27 score.

We created a web tool for obtaining individualized predictions. This tool allows users to input patient characteristics and provides outcome predictions over time. Figure 4 illustrates likely events through 60 months postbaseline for 3 hypothetical patients. The first patient is older, is a former smoker with anemia and positive p16 status, and has cT1N0 (localized) disease. The predicted probabilities of dying with or without earlier recurrence are similar for this patient. In contrast, the second patient is younger with more advanced p16-negative cancer and is predicted to have a much higher probability of locoregional recurrence. The third patient also has more advanced disease, but he is more likely to experience a distant metastasis than a locoregional recurrence owing to high N classification. These plots show how the likely outcome events can differ for patients with varying characteristics (eFigure 9 and eAppendix 7 in the Supplement provides details).

## Discussion

With advances in precision medicine, there is potential for improvement in prognostic modeling through incorporation of robust biomarkers along with traditional clinical features. In OPSCC, the AJCC 8th edition staging system incorporated p16 status to improve staging.^3^ Prognostic modeling can further personalize risk prediction. Patient-centered treatment decisions and counseling for individuals diagnosed with OPSCC rely on accurate prognostic estimates.

When developing a prognostic calculator, many risk factors can be included, even if each effect size is small. Multiple factors related to different posttreatment outcomes (eg, recurrence, other cause death) can be incorporated to produce individualized predictions. There are clear disadvantages to excluding known risk factors, including reduced prognostic accuracy and biased predictions.^41,42,43^ In addition, bias can result from inappropriate statistical techniques in the presence of competing risks or covariate-dependent censoring.^41^

Previous work^11,15,16^ has evaluated existing prognostic calculators for head and neck cancer^9,12,14,44^ to identify limitations and explore ways to develop a more clinically useful calculator. These studies found considerable variability in predictions for individual patients as well as discrepancies in calibration and AUC across calculators. Many existing calculators do not account for other causes of death and do not differentiate between different types of recurrence.

We developed a prognostic calculator informed by clinical, oncologic, and imaging data, resulting in good discrimination for overall survival surpassing that of the AJCC 8th edition overall cancer stage. This model is different in that it (1) considers multiple outcomes, (2) separates locoregional recurrence from distant metastases, and (3) incorporates imaging biomarkers from computed tomography and fluorodeoxyglucose positron emission tomography acquired during standard-of-care pretreatment workup. Providing personalized predictions of multiple outcomes increases the clinically useful information available for both patients and clinicians. This information may have clinical implications affecting additional treatment considerations and/or trial candidacy. For example, if the risk of recurrence within the patient’s expected lifetime is determined to be very low, they may be a good candidate for a de-escalation trial. Similarly, a high recurrence risk may influence the decision for intensified therapy.^45^ In addition, patients at high risk for dying from a condition unrelated to their disease may benefit from closer follow-up with a primary care physician to address comorbidities. To better inform these decisions, it is important that prognostic calculators account for risk factors associated with death from other causes. For our model, such factors include age, comorbidities score, and smoking status. For a prognostic calculator to be clinically useful, the input factors must be easily and commonly obtained. Biomarkers must be measured in a standardized fashion and routinely collected in clinical practice. We chose to include 2 imaging markers that are easily obtainable from standard-of-care pretreatment positron emission tomography and computed tomography scans. We further provide a version of the calculator that does not rely on these imaging biomarkers for use when these markers are unavailable.

To achieve the accuracy and clinical relevance necessary for this prognostic calculator to be useful, our group developed and applied novel statistical methods. Missing data of multiple types presented a challenge, and we developed imputation strategies for handling missingness in a principled way. The multistate model structure allowed us to consider multiple different outcomes, each of which may be influenced by different risk factors. In addition, this model incorporated structural features of OPSCC prognosis and accounted for competing risks. Bayesian estimation techniques and context-driven order restrictions enabled improved parameter estimation, allowing a wide spectrum of patient information to inform predictions.

Interaction with our application can aid in communicating prognosis to patients and may help inform medical decisions for practitioners. Future work will explore strategies for improving communication of predictions to patients and clinicians. We hope to include treatment modalities in future models, which will require consideration of known morbidities of various treatment modalities and must account for the interaction of treatment with other variables. Our group will work toward developing a production-level calculator to augment patient-centered decision-making for newly diagnosed OPSCC.

### Limitations

The study had limitations. External validation of this prognostic calculator indicated underestimation of mortality without a previous recurrence in patients from Erasmus MC. Several factors may contribute. Because our model does not adjust for treatment, treatment differences between the 2 cohorts could impact validation. Adult Comorbidity Evaluation score may not fully account for treatment-related comorbidities associated with higher other-cause mortality.^45,46^ In addition, the UM and Erasmus MC patients differed considerably in terms of smoking habits (approximately 65% current or former smokers in UM vs approximately 90% current or former smokers in Erasmus MC) and p16 positivity (50.6% in UM vs 18.8% in Erasmus MC), and baseline rates of other-cause mortality may also differ.

## Conclusions

In this study, we developed a prognostic calculator informed by clinical, oncologic, and imaging data, resulting in good discrimination for overall survival in training and validation cohorts. Interaction with our web application may aid in communicating prognosis to patients and informing medical decisions for practitioners.

## Notes

Supplement. **eAppendix 1.** Multistate Model Specification **eAppendix 2.** Prior Distributions and Estimation **eAppendix 3.** Imputation of Missing Data **eAppendix 4.** Obtaining Individualized Predictions **eAppendix 5.** Model Evaluation in University of Michigan Data **eAppendix 6.** Model Evaluation in Erasmus Validation Cohort **eAppendix 7.** Characterizing Each Variable’s Impact on Predictions
eReferences
**eTable 1.** Covariates Included in Each Component of the Multistate Model **eTable 2.** Observed State Transitions During Follow-up by Initial State in Data From The University of Michigan and Erasmus University Medical Center **eTable 3.** Prior Distributions for Model Parameters **eTable 4.** Assumed Parameter Ordering Incorporated Into Prior Distributions **eTable 5.** Posterior Means for Baseline Hazard Parameters and Intercepts **eTable 6.** Descriptives for Additional Covariates Used for Imputation **eTable 7.** Comparison of Discrimination of 5-Year Overall Survival Predictions With Predictions From Other Models in the Literature and With Known Risk Factors **eFigure 1.** Comparison of 5-Year Overall Survival Predictions Between Model With and Without Imaging Variables (rECE and MTV) **eFigure 2.** Evaluation of 5-Year Overall Survival and Event-Free Probabilities in Model Without Imaging Variables **eFigure 3.** Comparison of 5-Year Overall Survival Predictions (Model Without Imaging Variables) With Predictions From Other Models in the Literature **eFigure 4.** Evaluation of Overall Survival and Event-free Probabilities Over Time in Model Without Imaging Variables **eFigure 5.** Discrimination and Stratification of Observed Overall Survival by Predicted 5-Year Overall Survival Probabilities and T Classification **eFigure 6.** Evaluation of Individualized Predictions for 5-Year Overall Survival and Event-Free Survival From Model Without Imaging Variables in Independent Erasmus MC Validation Cohort **eFigure 7.** Evaluation of Average Predictions Across Patients (Shaded Regions) With Observed Outcomes (Points) in Erasmus MC Cohort **eFigure 8.** Cox-Snell Diagnostic Plots for Overall Survival and Event-Free Survival at 5 Years in Erasmus MC Cohort **eFigure 9.** Relative Influence of Various Patient Characteristics on Calculator Predictions of Overall Survival and Event-free Survival
