# Supplementary material for: Development and Assessment of a Model for Predicting Individualized Outcomes in Patients With Oropharyngeal Cancer
Source: JAMA Netw Open. 2021 Aug 9;4(8):e2120055. doi: 10.1001/jamanetworkopen.2021.20055 (PMC8353539; doi:10.1001/jamanetworkopen.2021.20055)
Supplement: Supplement. — eAppendix 1. Multistate Model Specification eAppendix 2. Prior Distributions and Estimation eAppendix 3. Imputation of Missing Data eAppendix 4. Obtaining Individualized Predictions eAppendix 5. Model Evaluation in University of Michigan Data eAppendix 6. Model Evaluation in Erasmus Validation Cohort eAppendix 7. Characterizing Each Variable’s Impact on Predictions eReferences eTable 1. Covariates Included in Each Component of the Multistate Model eTable 2. Observed State Transitions During Follow-up by Initial State in Data From The University of Michigan and Erasmus University Medical Center eTable 3. Prior Distributions for Model Parameters eTable 4. Assumed Parameter Ordering Incorporated Into Prior Distributions eTable 5. Posterior Means for Baseline Hazard Parameters and Intercepts eTable 6. Descriptives for Additional Covariates Used for Imputation eTable 7. Comparison of Discrimination of 5-Year Overall Survival Predictions With Predictions From Other Models in the Literature and With Known Risk Factors eFigure 1. Comparison of 5-Year Overall Survival Predictions Between Model With and Without Imaging Variables (rECE and MTV) eFigure 2. Evaluation of 5-Year Overall Survival and Event-Free Probabilities in Model Without Imaging Variables eFigure 3. Comparison of 5-Year Overall Survival Predictions (Model Without Imaging Variables) With Predictions From Other Models in the Literature eFigure 4. Evaluation of Overall Survival and Event-free Probabilities Over Time in Model Without Imaging Variables eFigure 5. Discrimination and Stratification of Observed Overall Survival by Predicted 5-Year Overall Survival Probabilities and T Classification eFigure 6. Evaluation of Individualized Predictions for 5-Year Overall Survival and Event-Free Survival From Model Without Imaging Variables in Independent Erasmus MC Validation Cohort eFigure 7. Evaluation of Average Predictions Across Patients (Shaded Regions) With Observed Outcomes (Points) in Erasmus MC Cohort eFigure 8 [file jamanetwopen-e2120055-s001.pdf]

Supplementary Online Content

Beesley LJ, Shuman AG, Mierzwa ML, et al. Development and assessment of a model for predicting individualized outcomes in patients with oropharyngeal cancer. *JAMA Netw Open*. 2021;4(8):e2120055. doi:10.1001/jamanetworkopen.2021.20055

eAppendix 1. Multistate Model Specification

eAppendix 2. Prior Distributions and Estimation

eAppendix 3. Imputation of Missing Data

eAppendix 4. Obtaining Individualized Predictions

eAppendix 5. Model Evaluation in University of Michigan Data

eAppendix 6. Model Evaluation in Erasmus Validation Cohort

eAppendix 7. Characterizing Each Variable's Impact on Predictions

eReferences

eTable 1. Covariates Included in Each Component of the Multistate Model

eTable 2. Observed State Transitions During Follow-up by Initial State in Data From The University of Michigan and Erasmus University Medical Center

eTable 3. Prior Distributions for Model Parameters

eTable 4. Assumed Parameter Ordering Incorporated Into Prior Distributions

eTable 5. Posterior Means for Baseline Hazard Parameters and Intercepts

eTable 6. Descriptives for Additional Covariates Used for Imputation

eTable 7. Comparison of Discrimination of 5-Year Overall Survival Predictions With Predictions From Other Models in the Literature and With Known Risk Factors

eFigure 1. Comparison of 5-Year Overall Survival Predictions Between Model With and Without Imaging Variables (rECE and MTV)

eFigure 2. Evaluation of 5-Year Overall Survival and Event-Free Probabilities in Model Without Imaging Variables

eFigure 3. Comparison of 5-Year Overall Survival Predictions (Model Without Imaging Variables) With Predictions From Other Models in the Literature

eFigure 4. Evaluation of Overall Survival and Event-free Probabilities Over Time in Model Without Imaging Variables

eFigure 5. Discrimination and Stratification of Observed Overall Survival by Predicted 5-Year Overall Survival Probabilities and T Classification

eFigure 6. Evaluation of Individualized Predictions for 5-Year Overall Survival and Event-Free Survival From Model Without Imaging Variables in Independent Erasmus MC Validation Cohort

eFigure 7. Evaluation of Average Predictions Across Patients (Shaded Regions) With Observed Outcomes (Points) in Erasmus MC Cohort

eFigure 8. Cox-Snell Diagnostic Plots for Overall Survival and Event-Free Survival at 5 Years in Erasmus MC Cohort

eFigure 9. Relative Influence of Various Patient Characteristics on Calculator Predictions of Overall Survival and Event-free Survival

© 2021 Beesley LJ et al. *JAMA Network Open.*

This supplementary material has been provided by the authors to give readers additional information about their work.

© 2021 Beesley LJ et al. *JAMA Network Open.*

eAppendix 1. Multistate Model Specification

In this section, we provide additional details related to the multistate model specification in **Figure 1** (reproduced below):

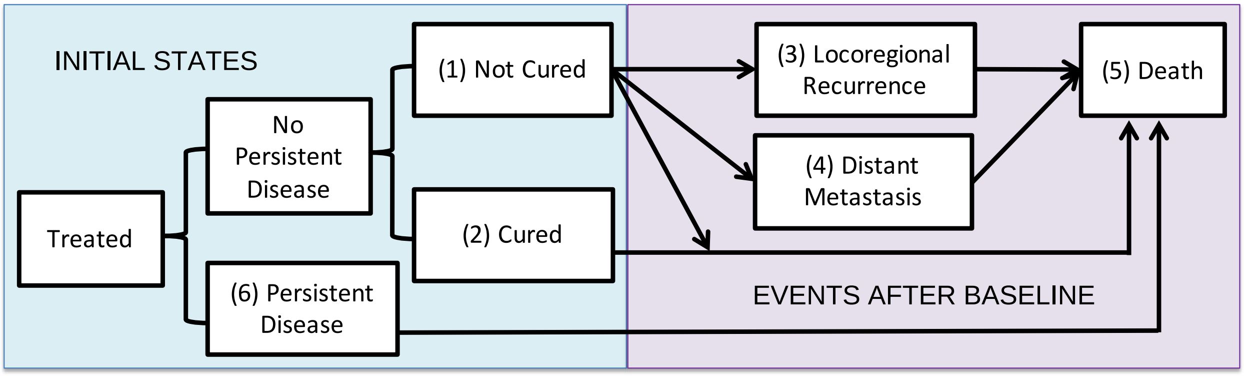

<u>Defining Initial States</u>

Let G represent a patient's initial (post-treatment) state. We define initial state G to take the following values:

G=1: patient is not cured and also not persistent
G=2: patient is cured of their primary cancer
G=6: patient has persistent disease after treatment

The value G takes is not usually known at the time of treatment, but we will suppose that it takes some fixed, unknown value. For some patients, the value of G can be determined based on what happens during follow-up. In our University of Michigan data, persistence status (whether G=6 or not) is not usually known at the time of treatment. However, disease persistence becomes apparent within the first 12 weeks after treatment, either through scans by 12 weeks after treatment to evaluate treatment response or through other indicators during routine follow-up in the first 12 weeks after treatment. For a small number of patients with less than three months clinical follow-up, persistence was defined based on whether or not they died within 6 months, where patients dying within 6 months of diagnosis were assumed to have persistent disease. Therefore, persistence status ($G = 6$ or $G \neq 6$) is known for all patients in our dataset by the end of follow-up.

Among the patients determined to be non-persistent ($G \neq 6$), the possible values of the initial state are 1 (not cured and not persistent) or 2 (cured). Non-persistent patients with observed recurrence during follow-up are known to have G=1 as their initial state. Additionally, we assume patients followed for at least 72 months without observed recurrence were in the "cured" initial state (G=2). For the remaining 50.2% of patients in our University of Michigan dataset who did not have persistent disease, did not have an observed recurrence, and were followed for recurrence for less than 72 months, initial state G is unknown and can be viewed as missing data. We will call this missingness in initial state G **Missingness Type I** in **eAppendix 3**.

© 2021 Beesley LJ et al. *JAMA Network Open.*

Defining Outcome Events

In our model, we consider three types of outcome events (**measured in months**): locoregional recurrence (without prior metastasis), distant metastasis (without prior locoregional recurrence), and death. Let T_r be the underlying recurrence time (first recurrence of either type), and let T_d be the underlying death time. In practice, these times may not always be observed, and we define T_r to be infinite for cured and persistent patients. In our model structure, therefore, only non-cured and non-persistent patients (G=1) have the potential to have a recurrence event.

We observe a censored version of our target outcome variables. Let Y_d be the observed death time or the censoring time for death (whichever is first), and let δ_d indicate whether death was observed. Let Y_r be the underlying recurrence time (first recurrence of either type) or the censoring time for recurrence (whichever is first). Let δ_r indicate whether recurrence was censored ($\delta_r = 0$) or whether a locoregional recurrence ($\delta_r = 1$) or distant metastasis ($\delta_r = 2$) was observed. For some patients (n=12), we know *that* a recurrence occurred and *when* it occurred, but we do not know whether $\delta_r = 1$ or $\delta_r = 2$. We call this missingness in type of recurrence **Missingness Type II** in **eAppendix 3**.

For now, we will assume that recurrence and death have the same censoring mechanism (i.e. we follow for recurrence as long as we follow for death for all patients, with $\delta_r = 0$ implying $Y_r = Y_d$). In reality, we have some patients (n=248) for whom recurrence is censored at some time $Y_r^0 < Y_d$, and for these patients, recurrence status and/or recurrence time is unknown during the time interval $[Y_r^0, Y_d]$. We call this missingness due to unequal censoring times for recurrence and death **Missingness Type III** in **eAppendix 3**. We describe an imputation strategy we use to handle unequal censoring times for the University of Michigan data later on (in **eAppendix 3**).

Model Structure

Let X represent the set of covariates (e.g. p16 status, T classification, etc.) that will be included in the multistate model. X may have missing values, called **Missingness Type IV** in **eAppendix 3**. We will use a different set of covariates for each component model of the multistate model described below, but we will describe the various component models below using the same set of covariates in each model for notational convenience. The multistate model as a whole consists of 8 component models: two logistic regression models related to the initial states and 6 Cox proportional hazards regression models related to the rate of different types of events after treatment (given initial state).

First, we model the initial state G using two logistic regression models as follows:

Component 1, probability of have persistent disease (G=6 vs. G=1 or 2):

$$logit\big(P(G = 6|X)\big) = \gamma_0 + \gamma_X X$$

Component 2, probability of being non-cured given non-persistent (G=1 vs. G=2 given G≠6)

$$logit\big(P(G = 1|X, G \neq 6)\big) = \alpha_0 + \alpha_X X$$

To describe events after baseline, we construct the multistate model in terms of relationships between different underlying states. Locoregional recurrence, distant metastasis, and death are

denoted as states 3, 4, and 5 respectively. Let $\lambda_{jk}(t)$ represent the hazard of transitioning between states j and k at time t. We model these transition hazards using the following proportional hazards regression model structures:

Component 3, locoregional recurrence among non-cured, non-persistent patients (G=1):
$$State\ 1 \rightarrow 3: \quad \lambda_{13}(t) = \lambda_{13}^{0}(t)e^{\beta_{13}X}$$
Component 4, distant metastasis among non-cured, non-persistent patients (G=1):
$$State\ 1 \rightarrow 4: \quad \lambda_{14}(t) = \lambda_{14}^{0}(t)e^{\beta_{14}X}$$
Component 5, death without prior recurrence for non-persistent patients (G=1 or G=2):
$$State\ 1 \rightarrow 5\ and\ 2 \rightarrow 5: \quad \lambda_{15}(t) = \lambda_{15}^{0}(t)e^{\beta_{15}X} = \lambda_{25}(t)$$
Component 6, death for persistent patients (G=6):
$$State\ 6 \rightarrow 5: \quad \lambda_{65}(t) = \lambda_{65}^{0}(t)$$

where $\lambda_{jk}^{0}(t)$ represents the baseline hazard for the transition between states j and k. Here, we do not include covariate effects in the transition to death among persistent patients (**Component 6**, states 6→5). This is because this event tends to happen quickly, and we do not have many persistent patients in our dataset to support estimation of covariate effects for that transition. In order to improve our ability to estimate model parameters, we assume that transitions to death without prior recurrence for cured and non-cured non-persistent patients (2→5 and 1→5) are the same (so $\lambda_{15}(t) = \lambda_{25}(t)$ as in **Component 5**). This same assumption is common for multistate models incorporating a cured initial state, as discussed in Conlon et al. (2013) and Beesley et al. (2018).[1,2]

When modeling death rates after either locoregional recurrence or distant metastasis, we use a clock-reset approach, where time is reset back to zero upon entering the recurrence state. We assume this model structure because cancer recurrence often triggers additional treatments, effectively starting off a new phase of patient care. These two transitions are modeled as follows:

Component 7, death after locoregional recurrence (without or without subsequent metastasis):
$$State\ 3 \rightarrow 5: \quad \lambda_{35}(t - T_r) = \lambda_{35}^{0}(t - T_r)e^{\beta_{35}X}, t > T_r$$
Component 8, death after metastasis (without or without subsequent locoregional recurrence):
$$State\ 4 \rightarrow 5: \quad \lambda_{45}(t - T_r) = \lambda_{45}^{0}(t - T_r)e^{\beta_{45}X}, t > T_r$$

In **Components 3-8**, event rates are a function of covariates through the β parameters and are functions of the time since baseline through the baseline hazard functions between states, $\lambda_{jk}^{0}(t)$.

For **Components 5-8**, we model the *cumulative* baseline hazard functions $\Lambda_{jk}^{0}(t) = \int_0^t \lambda_{jk}^{0}(u)\, du$ using Weibull hazards as follows:
$$\Lambda_{jk}^{0}(t) = \left(\frac{t}{\upsilon_{jk}}\right)^{\rho_{jk}}$$

For **Components 3 and 4**, standard Weibull baseline hazards fit the data poorly. In response to additional exploration of event rates in the data, we specified the following piecewise Weibull baseline hazards for these transitions:

$$\Lambda_{jk}^{0}(t) = \begin{cases} \left(\frac{t}{\upsilon_{jk1}}\right)^{1} & t \leq 6 \text{ months} \\ \left(\frac{6}{\upsilon_{jk1}}\right)^{1} + \left(\frac{t-6}{\upsilon_{jk2}}\right)^{\rho_{jk}} & t > 6 \text{ months} \end{cases}$$

Following previous work on identifiability of cure models, this multistate model is identifiable and can be fit to data with long enough follow-up for recurrence and death (as is the case for our University of Michigan Data). The complete data likelihood for this model is as follows:

$$L(\theta) = \left[e^{-\Lambda_{25}(Y_d)}\lambda_{25}(Y_d)^{\delta_d}\right]^{G=2}$$
$$x\left[e^{-\Lambda_{13}(Y_d)-\Lambda_{14}(Y_d)-\Lambda_{15}(Y_d)}\lambda_{15}(Y_d)^{\delta_d}\right]^{G=1,\delta_r=0}$$
$$x\left[e^{-\Lambda_{13}(Y_r)-\Lambda_{14}(Y_r)-\Lambda_{15}(Y_r)}\lambda_{13}(Y_r)\, e^{-\Lambda_{35}(Y_d-Y_r)}\lambda_{35}(Y_d-Y_r)^{\delta_d}\right]^{G=1,\delta_r=1}$$
$$x\left[e^{-\Lambda_{13}(Y_r)-\Lambda_{14}(Y_r)-\Lambda_{15}(Y_r)}\lambda_{14}(Y_r)\, e^{-\Lambda_{45}(Y_d-Y_r)}\lambda_{45}(Y_d-Y_r)^{\delta_d}\right]^{G=1,\delta_r=2}$$
$$x\left[e^{-\Lambda_{65}(Y_d)}\lambda_{65}(Y_d)^{\delta_d}\right]^{G=6}$$
$$x\,[P(G=6|X)]^{G=6}[1-P(G=6|X)]^{G\neq 6}$$
$$x\,[P(G=1|X,G\neq 6)]^{G=1}[1-P(G=1|X,G\neq 6)]^{G=2}$$

Inclusion of Covariates in Multistate Model

In applying this model to the head and neck cancer data, we use different sets of covariates in the different component models. Due to the large number of possible predictors, we did not include all predictors in all models and instead chose among the available predictors. For each transition, we chose covariates that we believed to be “important” or relevant to the corresponding model. For example, age, smoking status, and comorbidities would be important for modeling death without prior recurrence.

Inclusion of covariates in each component model is summarized in e**Table 1** below. In modeling whether or not a patient is persistent (**Component 1**), we included a small number of covariates, since there are not many persistent patients available to estimate parameters for this model. Additionally, cT classifications 1 through 3 were merged for this transition, since the majority of persistent patients had very high cT. Similarly, moderate and severe comorbidities were merged into a single parameter for the transitions to death after intermediate locoregional recurrence or distant metastasis due to the small number of patients in these categories with observed recurrences. In the model for the rate of metastasis (**Component 4**, states 1→4), we allow the effect of age to be different before or after age 65. This choice was made based on exploratory analyses that indicated different age associations among patients above and below roughly age 65.

We also consider two different model fits. In the second, we add biomarker information in the form of rECE and MTV. Due to small numbers of persistent and recurrent patients with observed biomarker values, biomarkers rECE and MTV were not included in the models for the probability of having persistent disease and for the models for death with or without prior recurrence.

A summary of the outcome events observed for the University of Michigan and Erasmus Medical Center cohorts can be found in e**Table 2**.

© 2021 Beesley LJ et al. *JAMA Network Open.*

eAppendix 2: Prior Distributions and Estimation

Prior Distributions

As part of the multistate model structure, we specify prior distributions for each of the model parameters. In e**Tables 3 and 4**, we provide information regarding the prior distributions assumed for this estimation.

The prior distributions for the Weibull baseline hazard parameters were chosen to allow a large range for the scale parameter (N(0,16) priors for log-scales) and were somewhat informative for the shape parameters (Gamma(2.4,0.4) priors). The Gamma hyperparameters were chosen to give a mean near 1, where a value of 1 for the shape parameter implies an exponential distribution for the corresponding event time.

For all covariate associations for Components 1-4, we used assumed wide N(0,4) priors (possibly with truncation to enforce order restrictions) for the log-hazard ratios and log-odds ratios. These fairly uninformative priors allow very strong covariate associations. More informative priors were chosen for Components 7-8. The rationale for this was two-fold: (1) less data was available for estimating these parameters, and more informative priors built in greater model stability and (2) we expect effects of baseline parameters to have weak associations with survival time, conditional on having experienced a recurrence.

Prior distributions for the intercepts of the two logistic regression models were normal distributions with prior means obtained based on preliminary descriptive statistics in the observed data, including the marginal proportion of patients experiencing persistence and the estimated proportion of patients who never experienced a recurrence obtained from Kaplan-Meier plotting of time to recurrence.

Estimation

Model parameters were estimated using a Markov Chain Monte Carlo (MCMC) algorithm, where at each iteration, each parameter was drawn one-by-one from its posterior distribution (a combination of its prior distribution and the likelihood from the multistate model of interest). Since the posterior distribution for each model parameter was complicated, we use the Metropolis-Hastings method to draw each model parameter using a normal random walk proposal distribution. For parameters with order restrictions, this proposal distribution is truncated to satisfy the order restrictions. Variance parameters for these proposal distributions were tuning parameters with values chosen to give somewhere between 20 and 80% acceptance rates and ensure good operating characteristics of the final MCMC parameter draws.

At the end of each MCMC iteration, missing data was filled in through imputation. Details about the missing data imputation strategies can be found in **eAppendix 3**. The MCMC algorithm was run for a total of 25,000 iterations, with the first 10,000 iterations removed as burn-in. The remaining 15,000 parameter draws were used to obtain posterior means for the parameters of interest. We use these posterior means to obtain individualized predictions as in **eAppendix 4**. 95% quantiles are obtained from these 15,000 iterations and used to construct 95% credible intervals for model parameters as presented in the main paper. The MCMC algorithm was written as custom software in R. Numerical integration performed as part of the missing data handling was performed using R package *cubature* (version 2.0.4), and the strategy for handling different censoring times for recurrence and death described in **eAppendix** 3 involved imputing

© 2021 Beesley LJ et al. *JAMA Network Open.*

recurrence times for some patients using the methods in Beesley et al. (2018) and R function *uniroot* in the *stats* package (version 3.6.3).

Posterior Means

In the main paper, we provide posterior means for all covariate associations in Components 1-8 of the multistate model. Here, we provide posterior means for baseline hazard parameters along with logistic regression intercepts in e**Table 5**.

© 2021 Beesley LJ et al. *JAMA Network Open.*

eAppendix 3: Imputation of Missing Data

We have missing data of four types as described in **eAppendix 1**. We handle each of these four types of missing data within the MCMC estimation algorithm through imputation. This involves drawing values of the missing data. Below, we provide details regarding how this imputation was performed for each type of missing data. In performing this imputation, we define the following variables:

Y_r^0: min(first recurrence event time, censoring time for recurrence)
δ_r^0: categorical variable for recurrence observed prior to censoring for recurrence
=0 for censored recurrence at time Y_r^0
=1 for locoregional recurrence at time Y_r^0
=2 for distant metastasis at time Y_r^0
=NA for missing *type* of recurrence at time Y_r^0

Y_d: min(death time, censoring time for death)
δ_d: binary indicator for whether death was observed prior to censoring for death
=0 for censored recurrence at time Y_d
=1 for locoregional recurrence at time Y_d

The above outcome variables correspond to the *observed* outcome data for each patient. We also perform each imputation step conditioning on the most recent imputed values for the other variables.

<u>**Missingness Type I**: Imputing initial state, G</u>
Initial state *G* was imputed for people with missing values using a distribution derived based on the multistate cure model structure similar to the expression in Beesley et al. (2018)[2] but extended to incorporate two intermediate recurrence states. Initial state *G* will be missing if and only if (1) we know $G \neq 6$ and (2) $\delta_r^0 = 0$ and $Y_r^0 < 72$.

We impute initial state *G* for patients missing *G* from a Bernoulli distribution with probability:

$$P(G = 1|X, G \neq 6, Y_r^0, \delta_r^0 = 0, Y_d, \delta_d) = \frac{Numerator}{Denominator}$$

$$Numerator = P(G = 1|G \neq 6, X)\lambda_{15}(Y_d)^{\delta_d} S_1(Y_d) + P(G = 1|G \neq 6, X)\, I(Y_r^0 < Y_d)\, y$$
$$Denominator = Numerator + P(G = 2|G \neq 6, X)\lambda_{25}(Y_d)^{\delta_d} S_2(Y_d)$$

Where $y = y_1 + y_2$ and

$$y_1 = \int_{Y_r^0}^{Y_d} \lambda_{13}(v) S_1(v) \lambda_{35}(Y_d - v)^{\delta_d} S_3(Y_d - v)\, dv$$
$$y_2 = \int_{Y_r^0}^{Y_d} \lambda_{14}(v) S_1(v) \lambda_{45}(Y_d - v)^{\delta_d} S_4(Y_d - v)\, dv$$

and we define functions

$$S_1(u) = e^{-\Lambda_{13}(u) - \Lambda_{14}(u) - \Lambda_{15}(u)}$$
$$S_3(u) = e^{-\Lambda_{35}(u)} \qquad S_4(u) = e^{-\Lambda_{45}(u)}$$

Missingness Type II: Imputing Cause of Known Recurrence

For some patients, we know that a recurrence occurred and when it occurred, but we don't know which type (locoregional recurrence or distant metastasis). We impute the probability that it is a locoregional recurrence as

$$P(\delta_r^0 = 1|X, Y_r^0, \delta_r^0 \neq 0, Y_d, \delta_d, G = 1) = \frac{\lambda_{13}(Y_r)S_1(Y_r)\lambda_{35}(Y_d - Y_r)^{\delta_d}S_3(Y_d - Y_r)}{\lambda_{13}(Y_r)S_1(Y_r)\lambda_{35}(Y_d - Y_r)^{\delta_d}S_3(Y_d - Y_r) + \lambda_{14}(Y_r)S_1(Y_r)\lambda_{45}(Y_d - Y_r)^{\delta_d}S_4(Y_d - Y_r)}$$

where Y_r^0 is the observed recurrence time.

Missingness Type III: Imputing Missing Recurrence Status and Time under Unequal Censoring of Recurrence and Death (G=1 only)

In handling imputation for Missingness Type III, we will define partially observed outcomes Y_r and δ_r to be the recurrence outcomes we *would have observed* if we had followed every patient for recurrence as long as we followed them for death. Relating these theoretical outcomes to observed data, we have

$$Y_r = \begin{cases} Y_r^0, \text{ all other cases} \\ NA \text{ but known to be in } (Y_r^0, Y_d], \quad \text{if } \delta_r^0 = 0 \text{ and } Y_r^0 < Y_d \text{ and } G = 1 \end{cases}$$

$$\delta_r = \begin{cases} 0, \quad \text{if } (\delta_r^0 = 0 \text{ and } Y_r^0 = Y_d) \text{ or } G \neq 1 \\ 1, \quad \text{if } \delta_r^0 = 1 \\ 2, \quad \text{if } \delta_r^0 = 2 \\ NA, \quad \text{if } \delta_r^0 = 0 \text{ and } Y_r^0 < Y_d \text{ and } G = 1 \end{cases}$$

These theoretical recurrence outcome variables are unobserved if and only if (1) $\delta_r^0 = 0$, (2) $Y_r^0 < Y_d$, and (3) $G = 1$. For these patients, we will apply a two-step procedure, where we first impute δ_r and then set Y_r to be equal to Y_d if we imputed δ_r=0 and impute an event time $Y_r \in (Y_r^0, Y_d]$ if we imputed δ_r=1 or 2. This is a generalization of the method in Beesley et al. (2018)[2] to handle the setting with two intermediate recurrence events rather than just one.

For people with missing values, we first impute δ_r from a multinomial distribution with the following probabilities (scaled to sum to 1).

$$P(\delta_r = 0|X, \delta_r^0 = 0, Y_r^0, Y_d, \delta_d, G = 1) \propto \lambda_{15}(Y_d)^{\delta_d}S_1(Y_d)$$
$$P(\delta_r = 1|X, \delta_r^0 = 0, Y_r^0, Y_d, \delta_d, G = 1) \propto y_1$$
$$P(\delta_r = 2|X, \delta_r^0 = 0, Y_r^0, Y_d, \delta_d, G = 1) \propto y_2$$

With y_1 and y_2 defined previously. For patients with an imputed recurrence ($\delta_r = 1$ or $\delta_r = 2$), we impute the timing of the recurrence from one of the following

$$f(T_r|\delta_r = 1, Y_r^0, Y_d, \delta_d, G = 1, T_r \text{ in } (Y_r^0, Y_d]) \quad \text{or} \quad f(T_r|\delta_r = 2, Y_r^0, Y_d, \delta_d, G = 1, T_r \text{ in } (Y_r^0, Y_d])$$

using methods we have developed previously in Beesley et al. (2018)[2] involving inversion of the corresponding survival functions.

© 2021 Beesley LJ et al. *JAMA Network Open.*

Missingness Type IV: Imputing Missing Covariate Values

For the covariate imputation, we use a chained equations-type approach called substantive model compatible fully conditional specification (SMC-FCS) described in Bartlett et al. (2014).[3] The goal of this imputation strategy is to allow for flexible imputation distributions for each covariate while ensuring that imputation distributions are compatible with the analysis model (i.e., the multistate model). The covariate imputation procedure involves imputing each covariate from its assumed distribution given all the other variables in the dataset. This distribution can be re-written as a function of the analysis model (the multistate cure model) and a model for that particular covariate given the other covariates (e.g., model for HPV status given age, comorbidities, etc.). Below, we provide additional details regarding how the covariate models were specified. Imputation of the covariates in the outcome model is improved by incorporating other auxiliary covariates, which are also imputed as part of the algorithm. **eTable 6** provides descriptives for other covariates incorporated into the imputation algorithm.

T classification 8th edition (1/2/3/4), multinomial regression
Covariate model includes HPV, age, N, smoking

N classification 7th edition (2a/b/c given N2), multinomial regression
Covariate model includes T, HPV, age, smoking

N classification 8th edition (0/1/2-3), multinomial regression
Covariate model includes T, HPV, age, smoking

Smoking (none/current/former), multinomial regression
Covariate model includes T, HPV, N, age, education, ACE27

ACE27 (none/mild/moderate/severe), multinomial regression
Covariate model includes smoking, HPV, age, education, gender, ECOG

P16 (negative/positive), logistic regression
Covariate Model includes sexual partners, age, gender, smoking status, HPV, T, N

Education (>HS [high school], HS or less), logistic regression
Covariate model includes HPV, gender, ACE27, smoking, age

ECOG (0/1+), logistic regression
Covariate model includes T, age, smoking and ACE27

Anemia (binary), logistic regression
Covariate model includes T, N, gender, age, and ACE27

Overall Cancer Stage 7th edition (I/II/III/IV), multinomial regression
Covariate model includes T, N, HPV, and age

Marital Status (Divorced/married/never married/separated/widowed), multinomial regression

© 2021 Beesley LJ et al. *JAMA Network Open.*

Covariate model includes HPV, age, gender, education, sexual partners

ECS [extracapsular spread] (no/yes), logistic regression

Covariate model includes age, T, N, HPV. All subjects with N = N0 are automatically given ECS = 'no'.

HPV [human papillomavirus] (negative/positive), logistic regression

Covariate model includes sexual partners, age, gender, smoking status, p16, N

Log(# Sexual partners + 1), continuous, linear regression

Covariate model includes HPV, gender, ACE27, smoking, age, education

Pack-years

Never smokers are given a cigarette packyear value of zero. Then, we make the assumption that patients that are current or former smokers have packyears > 0. This is not implied because the patients could have smoked something other than cigarettes. We model log-packyears using predictors HPV, gender, ACE27, current/former smoker, age, education.

Continuous variables imputed outside the range of the observed values are transformed to be on the edge of the range (e.g. cannot have imputed pack-years greater than the maximum observed pack-years value)

The model including log-MTV and RECE (0, 1/2) also involves imputation of these variables:

rECE (0/1+), logistic regression

Covariate model includes T, N, ACE27, matted node status, logMTV

Log(MTV), linear regression

Covariate model includes T, N, ACE27, logGTV, rECE

Log(GTV), linear regression

Covariate model includes logMTV, T, N

© 2021 Beesley LJ et al. *JAMA Network Open.*

eAppendix 4: Obtaining Individualized Predictions

In this section, we describe how we can use the multistate model structure to obtain state occupancy probabilities. These probabilities represent the proportion of patients with a given set of covariate values that would be in different outcome states at time t. We can estimate these probabilities across t to get a sense of the likelihood of different outcome events over time. Below, we provide formulas for calculating the probabilities, which can be viewed as cumulative incidence probabilities. Estimates of these various probabilities given a fixed covariate set X are provided in the online web tool.

First, we define the following probabilities:

$$p_6 = P(G = 6|X)$$
$$p_1 = P(G = 1|X) = P(G = 1|X, G \neq 6)[1 - P(G = 6|X)]$$
$$p_2 = P(G = 2|X) = [1 - P(G = 1|X, G \neq 6)][1 - P(G = 6|X)]$$

<u>State Occupancy Probabilities at time t</u>

Died after prior locoregional recurrence (with or without subsequent metastasis)

$$\pi_{1a}(t) = p_1 \int_0^t e^{-\Lambda_{13}(v) - \Lambda_{14}(v) - \Lambda_{15}(v)} \lambda_{13}(v) \left[1 - e^{-\Lambda_{35}(t-v)}\right] dv$$

Died after prior distant metastasis (with or without subsequent locoregional recurrence)

$$\pi_{1b}(t) = p_1 \int_0^t e^{-\Lambda_{13}(v) - \Lambda_{14}(v) - \Lambda_{15}(v)} \lambda_{14}(v) \left[1 - e^{-\Lambda_{45}(t-v)}\right] dv$$

Alive with prior locoregional recurrence (with or without subsequent metastasis)

$$\pi_{2a}(t) = p_1 \int_0^t e^{-\Lambda_{13}(v) - \Lambda_{14}(v) - \Lambda_{15}(v)} \lambda_{13}(v)\, e^{-\Lambda_{35}(t-v)} dv$$

Alive with prior distant metastasis (with or without subsequent locoregional recurrence)

$$\pi_{2b}(t) = p_1 \int_0^t e^{-\Lambda_{13}(v) - \Lambda_{14}(v) - \Lambda_{15}(v)} \lambda_{14}(v)\, e^{-\Lambda_{45}(t-v)} dv$$

Alive with no recurrence and not persistent

$$\pi_3(t) = p_1 e^{-\Lambda_{13}(t) - \Lambda_{14}(t) - \Lambda_{15}(t)} + p_2 e^{-\Lambda_{25}(t)}$$

Died without prior recurrence and not persistent

$$\pi_4(t) = p_1 \int_0^t e^{-\Lambda_{13}(v) - \Lambda_{14}(v) - \Lambda_{15}(v)} \lambda_{15}(v)\, dv + p_2[1 - e^{-\Lambda_{25}(t)}] dv$$

Alive with persistent disease

$$\pi_5(t) = p_6 e^{-\Lambda_{65}(t)}$$

Died with persistent disease

$$\pi_6(t) = p_6[1 - e^{-\Lambda_{65}(t)}]$$

We note that probabilities $\pi_3(t)$ and $\pi_4(t)$ could have been separated into contributions for cured (G=2) and non-cured (G=1) patients separately. However, it is well-known in cure model literature that the intercept related to the probability of being non-cured (here, the intercept from

© 2021 Beesley LJ et al. *JAMA Network Open.*

Component 2 in the multistate model) is the hardest to estimate. Therefore, we have greater confidence in our predictions of the merged survival outcomes without prior recurrence for cured and non-cured initial states than in the probabilities stratified by cure status. This is not a problem when separating out these probabilities for the persistent initial state (G=2 and probabilities $\pi_5(t)$ and $\pi_6(t)$) because persistence status is observed for all patients, and the corresponding intercept in **Component 1** is well-identified.

<u>Overall and Event-Free Survival</u>

Using these quantities, we can calculate the overall survival probability at time t as

$$OS(t) = \pi_{2a}(t) + \pi_{2b}(t) + \pi_3(t) + \pi_5(t)$$

and the event-free probability (with persistence counted as an event) as

$$EF(t) = \pi_3(t)$$

© 2021 Beesley LJ et al. *JAMA Network Open.*

eAppendix 5: Model Evaluation in University of Michigan Data

We calculate predictions of 5-year overall survival and 5-year event-free survival using our multistate models for all patients in our University of Michigan dataset. These predictions are based only on covariate information. For patients with missing covariate information, we calculate the prediction for each of 10 imputations of the covariates and average the resulting 5-year predictions.

We first compare the predictions for 5-year overall survival obtained from the models with and without imaging variables (MTV and rECE) in e**Figure 1**. For this figure, we only compare predictions for patients with *observed* imaging variables. We find that these predictions are generally close together, but there are some patients for whom the predictions differ substantially. In particular, the predicted survival probabilities tend to be lower for the model incorporating imaging data for patients with extracapsular expansion observed via imaging (rECE = 'yes').

Now, we seek to compare our predicted 5-year overall survival and event-free survival probabilities with observed data. One complication with evaluating observed event-free time is that some patients are censored for recurrence prior to censoring for death (**Missingness Type III**), and artificially censoring our event-free survival outcome accordingly could create bias. As a result, we evaluate our predictions using a single imputation of the recurrence information as discussed in **eAppendix 3**.

We now focus our attention on the model without the imaging variables. In e**Figure 2**, we evaluate how well our model-based predictions for 5-year overall survival and 5-year event-free survival can predict outcomes seen in our data in terms of calibration and discrimination. Unsurprisingly, these plots indicate good model calibration to the data used to fit the models. We see strong discrimination for both outcomes.

Previously, we published an article evaluating existing calculators of 5-year overall survival in the University of Michigan data. This previous analysis used a cohort of N=856, and the current paper considers data from a subset of N=840 of these patients. In developing this new calculator, we did extensive chart review to obtain additional recurrence information and to carefully evaluate persistence status. During this evaluation, several patients were determined not to meet our inclusion criteria, which requires patients to be newly diagnosed with oropharyngeal cancer and excludes patients with a second concurrent cancer, e.g. lung cancer. Here, we compare how our new model-based predictions compare to predictions from those existing calculators in the current cohort of N=840 patients. e**Figure 3** and e**Table 7** show the results.

We find that our proposed models tend to perform as well or better than existing calculators on our data (based solely on estimated AUC and C-Indices from the current cohort of N=840). The Denmark model also performs well in this cohort, producing a comparable AUC to the model without imaging biomarkers. Of course, we are evaluating our proposed models in the same data used to fit them, so these AUC and C-Index estimates will be overly optimistic.

Generally, our predictions tend to be highly correlated with other well-performing calculators (e.g. RTOG, Erasmus, Denmark) evaluated in Beesley et al. (2019).[4] We also present discrimination of age, 8th edition cT classification, and 8th edition overall cancer stage in e**Table 7**. We find that our model-based predictions provide much better discrimination than the single summary variables.

We use Cox-Snell diagnostic plots to evaluate how well the model predictions S(t) fit our observed data over time t. We calculate the predicted probability S(t) at the event/censoring time

for each patient. If the model fits well, 1-S(t) should have an approximately uniform distribution. The last row of e**Figure 4** shows the results, which indicate excellent predictions for both outcome events.

In addition to calibration and discrimination, we are also interested in how well our model predictions can stratify patients in terms of observed outcomes, where higher predicted survival probabilities should correspond to better overall survival. In e**Figure 5**, we calculate the Kaplan-Meier overall survival curve within strata defined by our model-based 5-year overall survival predictions for the models with and without imaging variables. For comparison, we also include a similar plot stratified by cT classification. We find that our 5-year overall survival predictions do a good job stratifying patients in terms of survival risk.

© 2021 Beesley LJ et al. *JAMA Network Open.*

eAppendix 6: Model Evaluation in Erasmus Validation Cohort

In this section, we evaluate how our model-based individualized predictions (model without imaging variables) perform in an independent validation dataset. We evaluate our model using data from 447 patients with oropharyngeal cancer treated at Erasmus MC in The Netherlands. **Table 1** provides descriptives of these patients. Investigators at Erasmus MC were not involved in the development of the statistical model. Code for generating model predictions based on baseline covariates was provided to the Erasmus group. Missing data were handled using MICE multiple imputation, and predictions were averaged across 15 imputed datasets.

Results are shown in e**Figures 6, 7, and 8**. We found generally good calibration properties for 5-year overall survival and event-free survival and reasonable discrimination. Calibration of overall survival estimates before 5 years was poor (e**Figure 7-8**), with model-predicted rates of death without prior recurrence being much lower than observed rates in the Erasmus MC data. This may be due to the very different demographics of the Erasmus cohort relative to the UM cohort. 78.3% of the Erasmus cohort reported being a current smoker, compared to only 31.9% of the UM cohort. The overall proportion of patients with positive p16 status was also strongly different between the two cohorts, with at least 50.6% of UM patients being p16 positive, compared to only 18.8% in the Erasmus cohort. These large demographic differences may translate into differing rates of other cause mortality that are not adequately captured by smoking status, age, and ACE27 comorbidities in our model for death without prior recurrence. Additionally, the Erasmus MC cohort is generally older (treatment 2000-2006) compared to the UM cohort (treatment 2003-2016), which may also contribute to differences in death rates without prior recurrence.

© 2021 Beesley LJ et al. *JAMA Network Open.*

eAppendix 7: Characterizing each variable's impact on predictions

Model-based predictions from **eAppendix 4** are complicated functions of the model parameters. **Figure 2** in the main paper provides point estimates and 95% credible intervals relating to each covariate's association in each component model of the larger multistate model. However, it can be difficult to understand the aggregate impact of each variable on predictions. In this section, we apply regression tree techniques to directly model the relationship between input covariates (age, T classification, smoking status, N classification, anemia status, p16 status, ACE267, rECE, MTV, and gender) on model-based predictions for overall survival and event-free survival. For different time horizons (3, 6, 10, 20 30, 40, 50, and 60 months post-diagnosis), we obtain our predictions for overall and event-free survival for each of the 840 patients. For each time horizon, we estimate the association between covariates and each of the predicted outcomes using random forest modeling using the *gbm* package in R. The goal of this modeling is to characterize the complicated non-linear relationship between each of the covariates and the proposed multistate model predictions. Restated, this analysis is an attempt to approximate and describe the mapping between each patient characteristic and our multistate model predictions.

Using these statistical methods, we can obtain a measure of the relative influence of each covariate on multistate model predictions, where influence is measured as the negative impact of randomly shuffling the given covariate on our ability to recover the model-based predictions. Higher relative influence values indicate a stronger association between that variable and the multistate model predictions. This association can vary for different time horizons, where cancer-related covariates may be very strongly related to mortality sooner after diagnosis and factors related to other cause mortality may be more strongly related to mortality long after diagnosis. e**Figure 9** shows the results. We generally find that the relative influence of age increases for later time horizons. Additionally, baseline T classification initially dominates event-free and overall survival rates soon after baseline. We should be careful not to over-interpret changes in the *magnitude* of the relative influence across different time horizons. Instead, we can focus on the *ordering* of the relative importance measure across time horizons, which can give us a sense of which covariate or covariates are dominating the predicted values at each time horizon. Expanded modeling using a larger range of input covariates may do a better job at characterizing the complicated relationship between inputs and outputs, and this exploration can be viewed as a *rough* summary of these variable relationships in our UM cohort.

© 2021 Beesley LJ et al. *JAMA Network Open.*

eReferences

1. Conlon, A. S. C., Taylor, J. M. G. & Sargent, D. J. Multi-state Models for Colon Cancer Recurrence and Death with a Cured Fraction. *Stat. Med.* **33**, 1750–1766 (2013).
2. Beesley, L. J. & Taylor, J. M. G. EM Algorithms for Fitting Multistate Cure Models. *Biostatistics* **20**, 416–432 (2018).
3. Bartlett, J. W., Seaman, S. R., White, I. R. & Carpenter, J. R. Multiple imputation of covariates by fully conditional specification: accomodating the substantive model. *Stat. Methods Med. Res.* **24**, 462–487 (2014).
4. Beesley, L. J. *et al.* Individualized Survival Prediction for Patients with Oropharyngeal Cancer in the Human Papillomavirus Era. *Cancer* **125**, 69–78 (2019).

eTable 1: Covariates included in each component of the multistate model

Model Covariates	Component Model					
	1	2	3	4	7 and 8	5
	Persistent (yes/no)	Non-cured (yes/no) given not persistent	Locoregional recurrence rate	Distant metastasis rate	Death rate after recurrence	Death rate without recurrence
Age (in decades after centering at 58.5)	Yes	Yes	Yes	Separate parameters before/after age 65	Yes	Yes
cT - T1, T2, T3, T4	cT1-3 merged	Yes	Yes	Yes	No	No
cN - N0/1, N2, N3	No	Yes	Yes	Yes	No	No
ACE27 comorbidities - none, mild, moderate, severe	moderate and severe merged	Yes	No	No	moderate and severe merged	Yes
Smoking status - none, current, former	No	Yes	No	No	Yes	Yes
P16 status - positive, negative	Yes	Yes	Yes	Yes	Yes	Yes
Gender - male, female	No	Yes	Yes	Yes	No	Yes
Anemia - no, yes	No	Yes	Yes	Yes	Yes	Yes
rECE - no, yes	No	Model 2 Only	Model 2 Only	Model 2 Only	No	No
Log(MTV) - centered at 2.5	No	Model 2 Only	Model 2 Only	Model 2 Only	No	No

© 2021 Beesley LJ et al. *JAMA Network Open.*

eTable 2: *Observed state transitions during follow-up by initial state in data from The University of Michigan and Erasmus University Medical Center*

		Observed events, n			
Initial model state	Total, n (%)	Locoregional recurrence[a]	Distant metastasis[a]	Recurrence (unknown type)	Death
The University of Michigan					
Persistent (State 6)	47 (5.6)	-	-	-	45
Not persistent and not cured (State 1)	185 (22.0)	75	98	12	144
Cured[b] (State 2)	186 (22.1)	-	-	-	15
Cure status unknown[c] (State 1 or 2)	422 (50.2)	-	-	-	68
Total, n (%)	840 (100)	75 (8.9)	98 (11.7)	12 (1.2)	272 (32.3)
Erasmus University Medical Center					
Persistent (State 6)	38 (8.5)	-	-	-	38
Not persistent and not cured (State 1)	114 (25.5)	68	46	0	103
Cured[b] (State 2)	96 (21.4)	-	-	-	17
Cure status unknown[c] (State 1 or 2)	199 (44.5)	-	-	-	152
Total, n (%)	447 (100)	68 (15.2)	46 (10.3)	0	310 (69.4)

[a] as first observed event [b] cured defined as no observed recurrence within 72 months
[c] not persistent and censored for recurrence prior to 72 months
- indices events that cannot occur under model assumptions

© 2021 Beesley LJ et al. *JAMA Network Open.*

eTable 3: Prior Distributions for Model Parameters

Parameter	Prior Distribution
Shape parameters for Components 3-8: ρ_{jk}	Gamma(2.4, 0.4)
Scale parameters for Components 3-8: $\log(\nu_{jk})$	Normal(0,16)[b]
Covariate parameters for Components 1-5: β_{13} , β_{14}, β_{15}, α_X, ω_X	Normal(0,4)[a]
Covariate parameters for Components 7-8: β_{35} , β_{45}	Normal(0,2)[a]
Intercept for Component 1: γ_0	Normal(-2.92,2)
Intercept for Component 2: α_0	Normal(-0.62,2)

[a] In the case of order restrictions, these are truncated normal distributions that satisfy the order restrictions [b] Normal(u,v): "u" corresponds to the mean and "v" corresponds to the variance.

© 2021 Beesley LJ et al. *JAMA Network Open.*

eTable 4: Assumed parameter ordering incorporated into prior distributions

Component Model	Ordered Predictors
Component 1:<br>P(persistent |X)	Higher odds with higher T-classification<br>- Parameter for cT4 > 0
Component 2:<br>P(not cured | not persistent, X)	Higher odds with higher T-classification<br>- Parameter for cT1 < cT2 < cT3 < cT4<br><br>Higher odds with higher N-classification<br>- Parameter for cN0/1 < cN2 < cN3
Component 3 (states 1→3):<br>Locoregional recurrence	none
Component 4 (states 1→4):<br>Distant metastasis	Higher hazard with higher N-classification<br>- Parameter for cN0/1 < cN2 < cN3
Component 5 (states 1 or 2 →5):<br>Death without prior recurrence	Higher hazard with older age<br>- Parameter for Age > 0<br><br>Higher hazard with worse smoking habits<br>- Parameter for Never smoker < former < current<br><br>Higher hazard with more comorbidities<br>- Parameter for ACE none < mild < moderate < severe
Component 7 (states 3→5):<br>Death after locoregional recurrence	none
Component 8 (states 4→5):<br>Death after distant metastasis	none

© 2021 Beesley LJ et al. *JAMA Network Open.*

eTable 5: Posterior means for baseline hazard parameters and intercepts[a]

Model: Parameter	Model without imaging variables	Model with imaging variables
Component 1 intercept: γ_0	-4.12	-4.09
Component 2 intercept: α_0	-2.29	-2.64
Component 3 baseline hazard: $\rho_{13}, \log(\upsilon_{131}), \log(\upsilon_{132})$	0.69, 4.61, 3.20	0.72, 4.87, 3.30
Component 4 baseline hazard: $\rho_{14}, \log(\upsilon_{141}), \log(\upsilon_{142})$	0.82, 5.02, 3.46	0.95, 5.70, 3.62
Component 5 baseline hazard: $\rho_{15}, \log(\upsilon_{15})$	1.04, 7.06	1.03, 7.00
Component 6 baseline hazard: $\rho_{65}, \log(\upsilon_{65})$	0.68, 2.31	0.67, 2.32
Component 7 baseline hazard: $\rho_{35}, \log(\upsilon_{35})$	0.71, 4.21	0.71, 4.17
Component 8 baseline hazard: $\rho_{45}, \log(\upsilon_{45})$	0.85, 3.56	0.87, 3.51

[a] here, “imaging variables” refers to inclusion of rECE and MTV

© 2021 Beesley LJ et al. *JAMA Network Open.*

eTable 6: Descriptives for additional covariates used for imputation

Characteristic	Value
Pack-years	
Mean (SD)	20.6 (25.4)
Unknown, n (%)	40 (4.8)
ECOG performance status, n (%)	
0	406 (48.3)
1 or 2	52 (6.2)
Unknown	382 (45.5)
Maximum level of education, n (%)	
HS or lower	162 (19.3)
> HS	376 (44.8)
Unknown	302 (36.0)
N-classification (7th edition), n (%)	
0/1	191 (22.7)
2	44 (5.4)
2a	64 (7.6)
2b	325 (38.7)
2c	138 (16.4)
3	78 (9.3)
Overall cancer stage (7th edition), n (%)	
I	15 (1.8)
II	35 (4.2)
III	96 (11.4)
IV	693 (82.5)
Unknown	1 (0.1)
HPV DNA, n (%)	
Negative	92 (10.9)
Positive	490 (58.3)
Unknown	258 (30.7)
Marital status, n (%)	
Divorced	118 (14.0)
Married	410 (48.8)
Never Married	32 (3.8)
Separated	11 (1.3)
Widowed	28 (3.3)
Unknown	241 (28.7)
Extracapsular spread (ECS), n (%)	
No	159 (18.9)
Yes	124 (14.8)
Unknown	557 (66.3)
Number sexual partners	
Median (SD)	10.0 (46.1)
Unknown, n (%)	597 (71.1)
Gross Tumor Volume (cc)	
Mean (SD)	83.3 (57.6)
Unknown, n (%)	447 (53.2)

© 2021 Beesley LJ et al. *JAMA Network Open.*

eTable 7: Comparison of discrimination of 5-year overall survival predictions with predictions from other models in the literature and with known risk factors

Details	Category	AUC	C-Index
Proposed Model without imaging	New model	0.78	0.76
Proposed Model with imaging[a]	New model	0.81	0.78
RTOG Model[b]	Existing model	0.73	0.74
Erasmus Model[b]	Existing model	0.71	0.73
Denmark Model[b]	Existing model	0.78	0.77
Age	Single variable	0.57	0.60
8th edition cT classification	Single variable	0.67	0.64
8th edition overall cancer stage	Single variable	0.71	0.68

[a] imaging variables include rECE and MTV

[b] Beesley et al. (2019) estimated AUC for the RTOG model was 0.77 (C-Index: 0.73), for the Erasmus model was 0.75 (C-Index: 0.74), and for the Denmark model was 0.80 (C-Index: 0.78) using a larger patient cohort, some patients of which were excluded in the current analysis.

© 2021 Beesley LJ et al. *JAMA Network Open.*

eFigure 1: Comparison of 5-year overall survival predictions between model with and without imaging variables (rECE and MTV).*

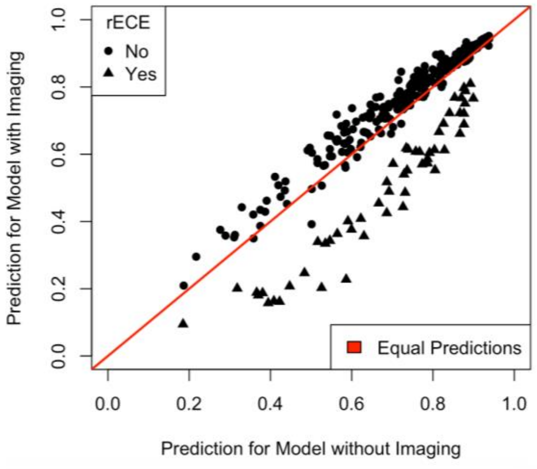

*predictions shown for 276 patients with observed MTV and rECE

© 2021 Beesley LJ et al. *JAMA Network Open.*

eFigure 2: Evaluation of 5-year overall survival and event-free probabilities in model without imaging variables*

5-Year Overall Survival	5-Year Event-free survival
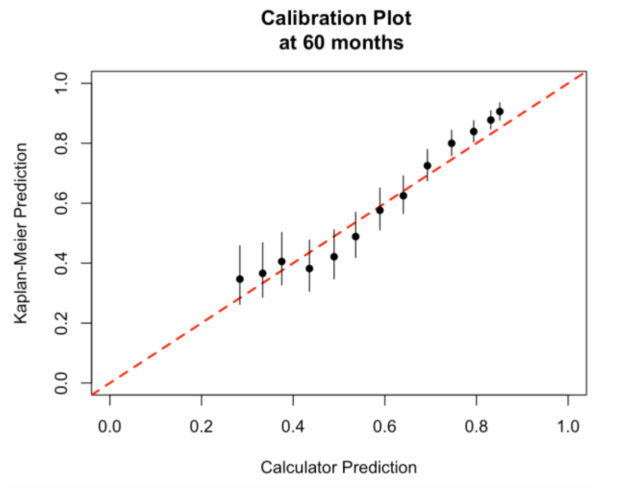	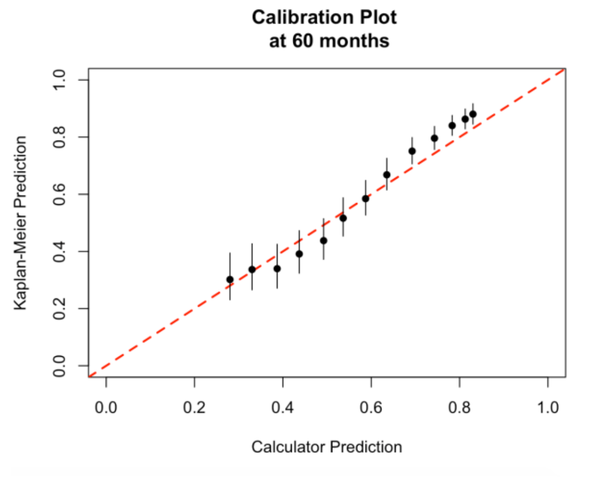
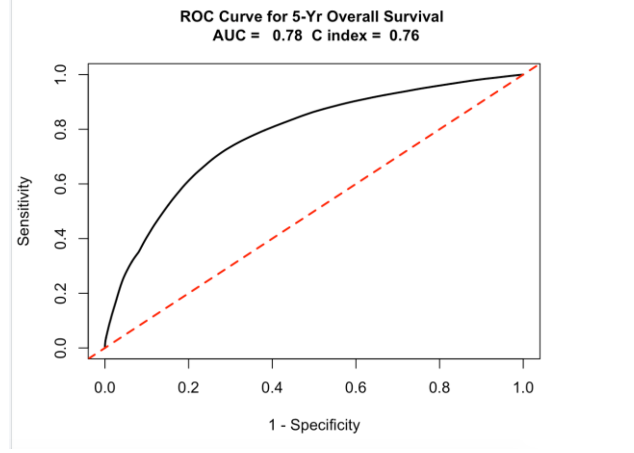	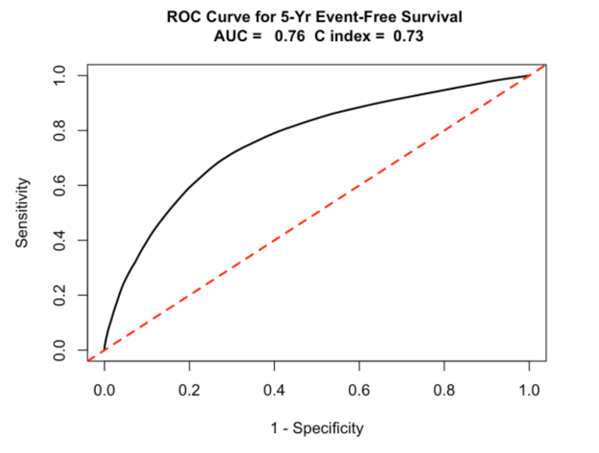

*In calibration plots, each point represents the KM estimate and 95% confidence interval (y-axis) within a moving window of predicted event probabilities (average on x-axis).

© 2021 Beesley LJ et al. *JAMA Network Open.*

eFigure 3: Comparison of 5-year overall survival predictions (model without imaging variables) with predictions from other models in the literature

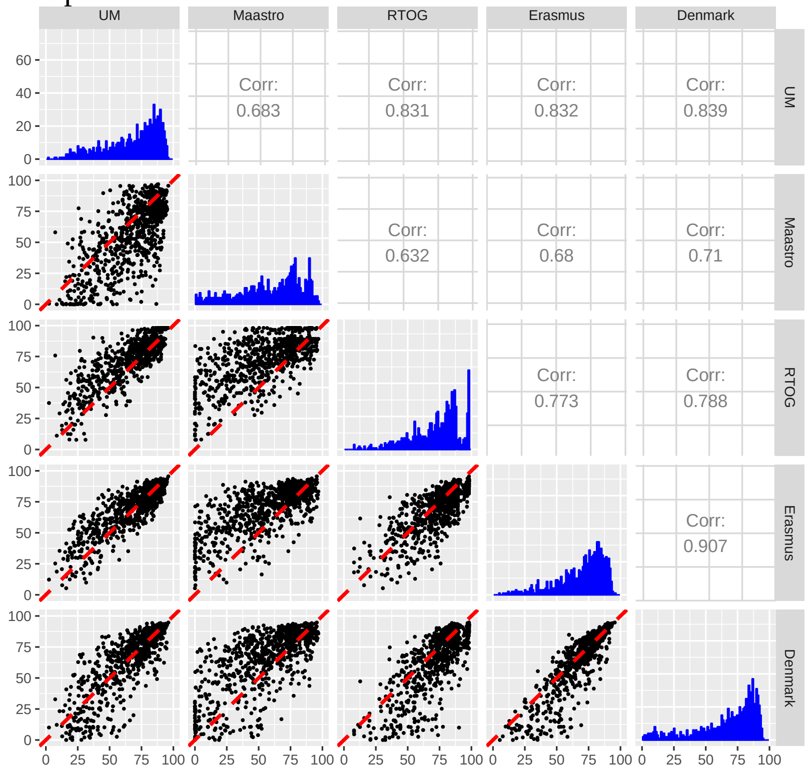

© 2021 Beesley LJ et al. *JAMA Network Open.*

eFigure 4: Evaluation of overall survival and event-free probabilities *over time* in model without imaging variables

<table>
<tr><th>Overall Survival</th><th>Event-free survival</th></tr>
<tr><td>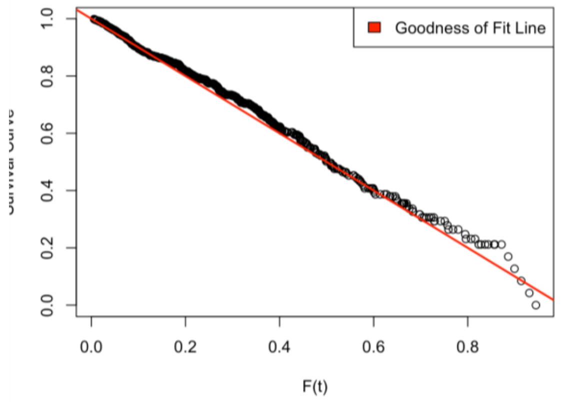
</td><td>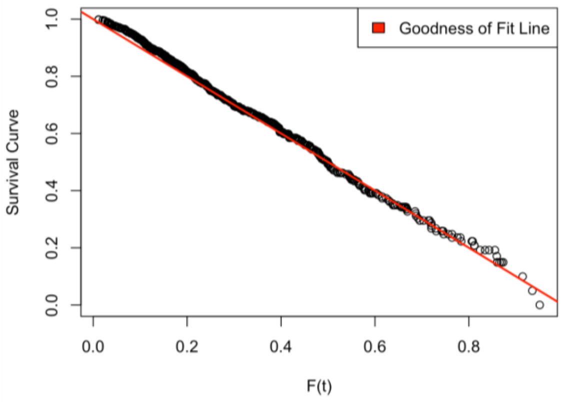
</td></tr>
</table>

© 2021 Beesley LJ et al. *JAMA Network Open.*

eFigure 5: *Discrimination and stratification of observed overall survival by predicted 5-year overall survival probabilities and T classification**

<table>
<tr><th>Predictions from Model without Imaging</th><th>Predictions from Model with Imaging</th><th>8th Edition T Classification</th></tr>
<tr><td>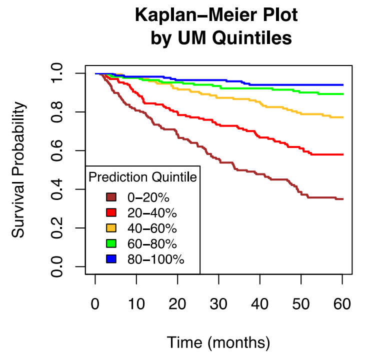
</td>
<td>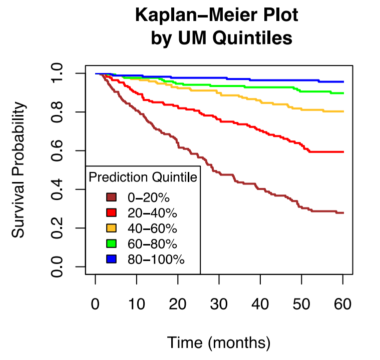
</td>
<td>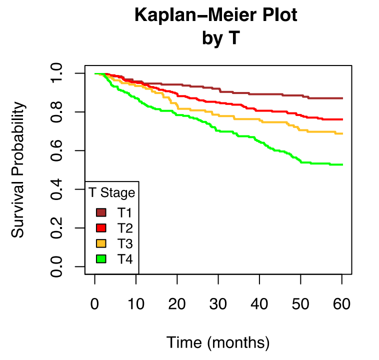
</td></tr>
</table>

*imaging variables include both rECE and MTV

© 2021 Beesley LJ et al. *JAMA Network Open.*

eFigure 6: Evaluation of individualized predictions for 5-year overall survival and event-free survival from model without imaging variables in independent Erasmus MC validation cohort*

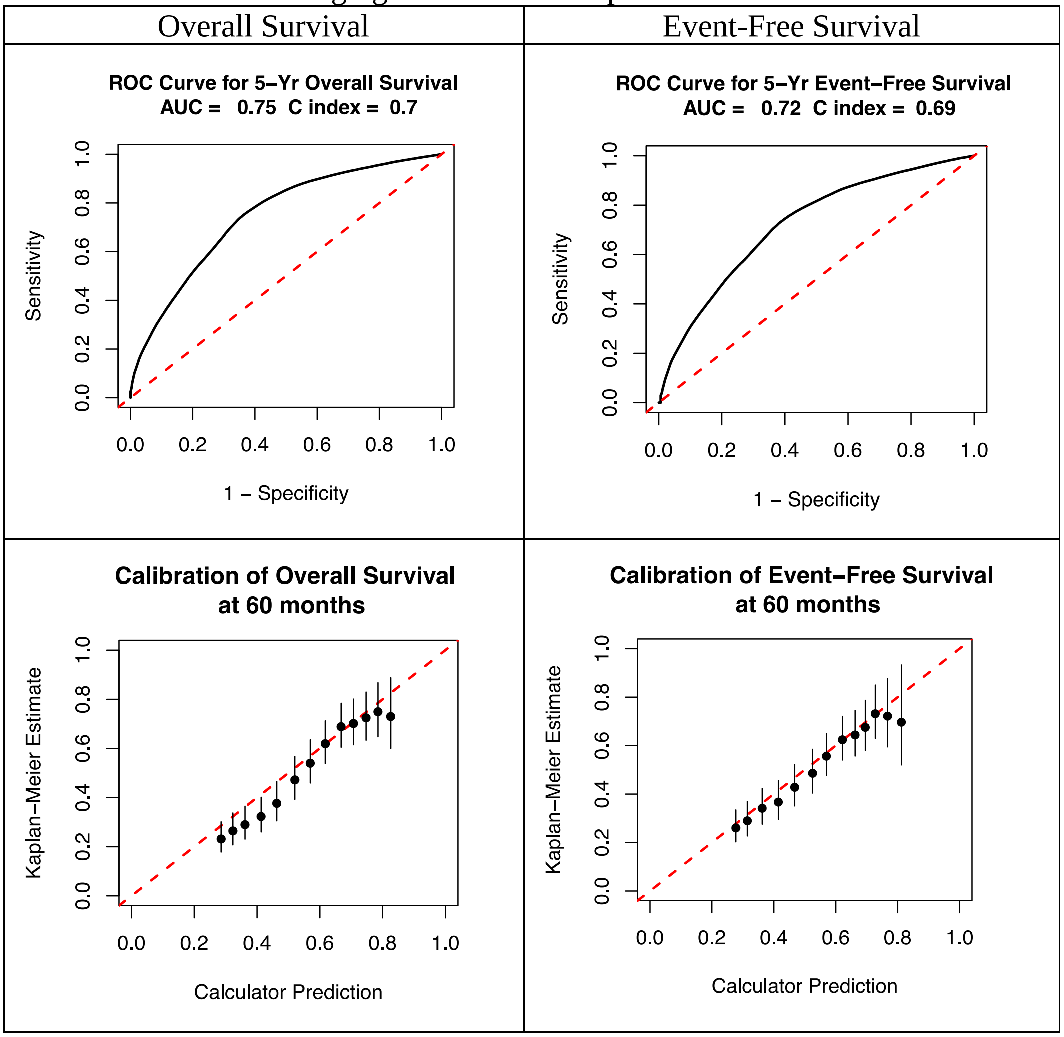

*In calibration plots, each point represents the KM estimate and 95% confidence interval (y-axis) within a moving window of predicted event probabilities (average on x-axis).

© 2021 Beesley LJ et al. *JAMA Network Open.*

eFigure 7: Evaluation of average predictions across patients (shaded regions) with observed outcomes (points) in Erasmus MC cohort*

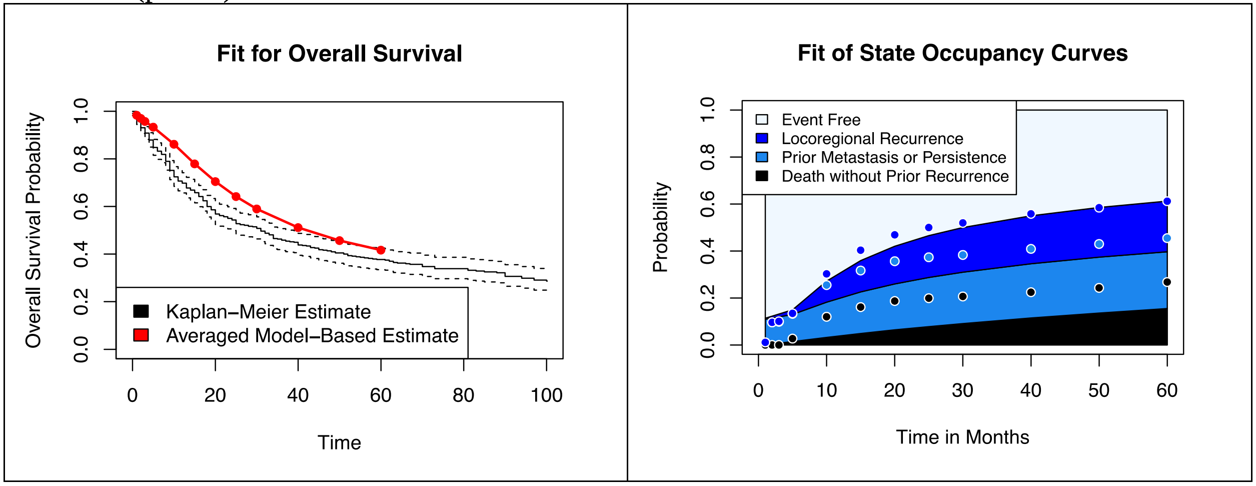

*Left: average predicted OS probabilities over time, plotted against Kaplan-Meier Estimate.
Right: Solid colors blocks indicate the average model-based prediction across Erasmus MC patients. These predictions are broken into four possible outcomes: (1) no event, (2) alive or died with prior metastasis as first event, (3) alive or died with prior locoregional recurrence as first event or with persistent disease, and (4) death without prior recurrence. By definition, these predicted probabilities sum to 1 at each time horizon. Plotted points indicate the corresponding cumulative incidence estimate for outcomes (1), (1) or (2), and (1) or (2) or (3) respectively, based on observed outcome data.

© 2021 Beesley LJ et al. *JAMA Network Open.*

eFigure 8: Cox-Snell diagnostic plots for overall survival (left) and event-free survival (right) at 5 years in Erasmus MC cohort*

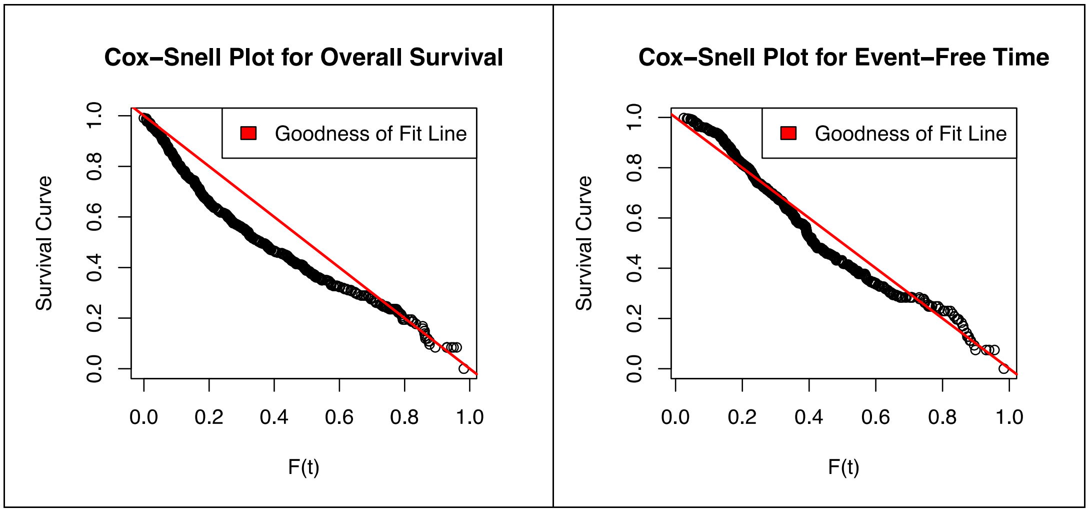

© 2021 Beesley LJ et al. *JAMA Network Open.*

eFigure 9: Relative influence of various patient characteristics on calculator predictions of (a) overall survival and (b) event-free survival*

(a) Overall survival

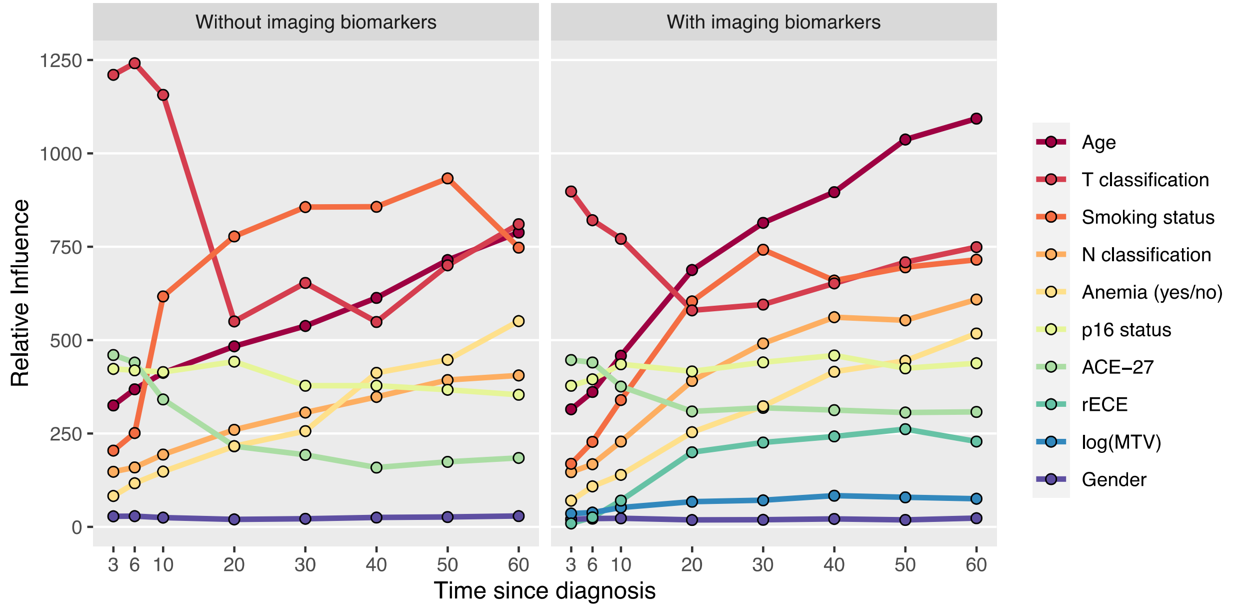

(b) Event-free survival

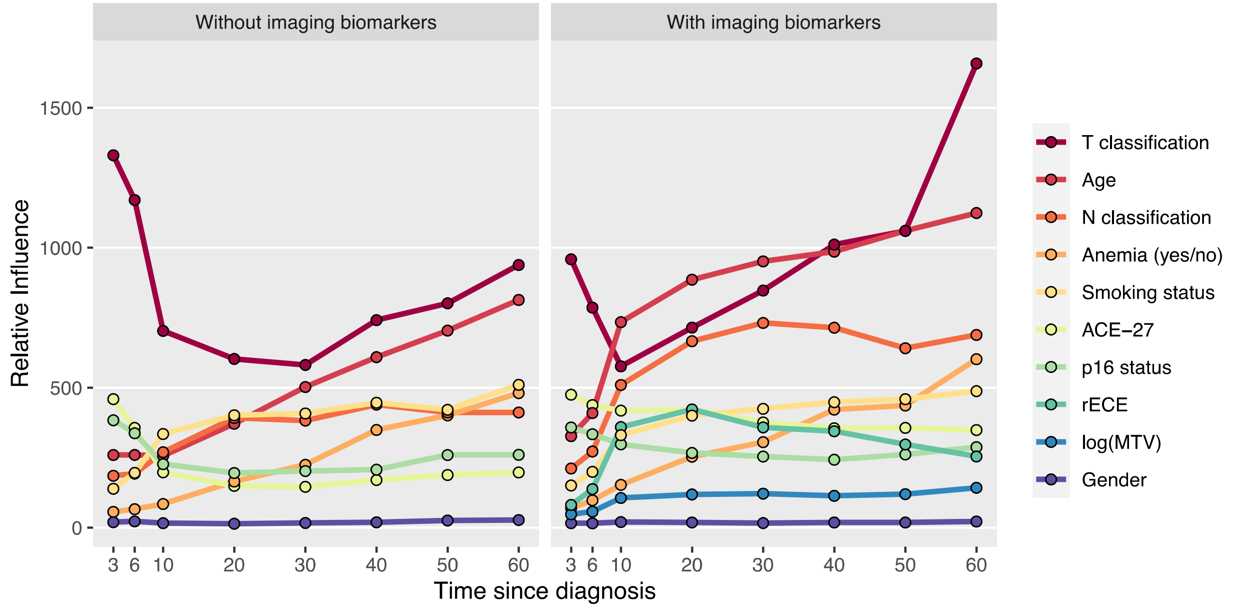

*The relative influence measures the increase in the prediction error (in this case, the ability of *the random forest* to predict *our calculator predictions*) when each variable is shuffled (permuted). Higher values indicate a stronger association between that variable and our calculator outputs.

© 2021 Beesley LJ et al. *JAMA Network Open.*
